# Efficacy and safety of antagonists for chemoattractant receptor-homologous molecule expressed on Th2 cells in adult patients with asthma: a meta-analysis and systematic review

**DOI:** 10.1186/s12931-018-0912-y

**Published:** 2018-11-09

**Authors:** Jing Yang, Jian Luo, Ling Yang, Dan Yang, Dan Wang, Bicui Liu, Tingxuan Huang, Xiaohu Wang, Binmiao Liang, Chuntao Liu

**Affiliations:** 1 Department of Respiratory and Critical Care Medicine, West China School of Medicine and West China Hospital, Sichuan University, No.37, Guoxue Alley, Chengdu, 610041 China; 2 Department of Respiratory Medicine, Mianyang Central Hospital, Mianyang, 621099 China

**Keywords:** Asthma, CRTH2 antagonist, Lung function, Adverse events, Meta-analysis, Systematic review

## Abstract

**Background:**

Chemoattractant receptor-homologous molecule expressed on Th2 cells (CRTH2) antagonists are novel agents for asthma but with controversial efficacies in clinical trials. Therefore, we conducted a meta-analysis to determine the roles of CRTH2 antagonists in asthma.

**Methods:**

We searched in major databases for RCTs comparing CRTH2 antagonists with placebo in asthma. Fixed- or random-effects model was performed to calculate mean differences (MD), risk ratio (RR) or risk difference (RD) and 95% confidence interval (CI).

**Results:**

A total of 14 trails with 4671 participants were included in our final analysis. Instead of add-on treatment of CRTH2 antagonists to corticosteroids, CRTH2 antagonist monotherapy significantly improved pre-bronchodilator FEV_1_ (MD = 0.09, 95% CI 0.04 to 0.15, *P* = 0.0005), FEV_1_% predicted (MD = 3.65, 95% CI 1.15 to 6.14, *P* = 0.004), and AQLQ (MD = 0.25, 95% CI 0.09 to 0.41, *P* = 0.002), and reduced asthma exacerbations (RR = 0.45, 95% CI 0.23 to 0.85, *P* = 0.01). Rescue use of SABA was significantly decreased in both CRTH2 antagonist monotherapy (MD = − 0.04, 95% CI -0.05 to − 0.03, *P* < 0.00001) and as add-on to corticosteroids (MD = − 0.78, 95% CI -1.47 to − 0.09, *P* = 0.03). Adverse events were similar between the intervention and placebo groups.

**Conclusions:**

CRTH2 antagonist monotherapy can safely improve lung function and quality of life, and reduce asthma exacerbations and SABA use in asthmatics.

**Electronic supplementary material:**

The online version of this article (10.1186/s12931-018-0912-y) contains supplementary material, which is available to authorized users.

## Introduction

Asthma is a common respiratory disease characterized by chronic airway inflammation, airway hyperresponsiveness, and reversible airflow limitation, which affects more than 300 million people worldwide and imposes a considerable social and economic burdens [1]. Most of patients can be effectively controlled by inhaled corticosteroids, the first-line therapy as recommended by the Global Initiative for Asthma (GINA) guideline [2], however, at least 40% of asthmatics remain inadequately controlled in spite of treatment with high dose of inhaled corticosteroids [3]. Moreover, a clear association between risk of adverse effects and long-term use of corticosteroid has also been observed [4], therefore, novel therapeutics is warranted to improve symptoms control and avoid overuse of steroids.

The chemoattractant receptor-homologous molecule expressed on Th2 cells (CRTH2) is a G-protein coupled receptor, and it is reported to be crucial in asthma development due to the chemotaxis of type 2 helper T cells and eosinophils, delay in cell apoptosis, as well as production of proinflammatory cytokines including interleukin-4, 5, and 13 by the activation of prostaglandin D_2_ (PGD_2_) [5–7]. Accumulating evidence has shown that the blockade of CRTH2 receptor significantly reduces allergic airway inflammation in animal models [8–10], but inconsistent efficacy and safety profiles of CRTH2 antagonists are noticed in clinical trials. Barnes and his colleagues [11] for the first time reported that OC000459, a CRTH2 antagonist, significantly improved quality of life but had no effect on lung function and airway inflammation in patients with asthma, while a significant improvement of forced volume in one second (FEV_1_) [12] and inhibition of post-allergen increase in sputum eosinophils [13] but no relief of asthma symptoms in symptomatic controller-naïve asthmatics [14] were reported by subsequent studies.

Based on the current controversial and ambiguous findings in the treatment of patients with asthma by CRTH2 antagonists, we conducted a meta-analysis and systematic review of all available randomized controlled trials (RCTs) to further determine the roles of CRTH2 antagonists in asthmatics.

## Methods

### Search strategies

A comprehensive computer search was conducted in Cochrane Central Register of Controlled Trials (CENTRAL), Pubmed, Medline, Embase, ISI Web of Science and American College of Physician (ACP) between 1946 and September 2018 by using the keywords of “CRTH2” or “chemoattractant receptor-homologous molecule expressed on TH2 cells” or “chemoattractant receptor expressed on TH2 cells” or “DP2” or “prostaglandin D2 receptor” and “antagonist” or “inhibitor” and “asthma”. Publication type and species were limited to RCTs and humans, respectively, but we did not limit the publication language. References listed in each identified article were checked and the related articles were searched manually to identify all eligible studies and minimize the potential publication bias.

### Inclusion and exclusion criteria

Eligible clinical trials were identified based on the following criteria: 1) asthma was diagnosed by physicians according to the GINA guideline [2] with the evidence of airway hyperresponsiveness (the provocation concentration of methacholine causing a 20% fall in FEV_1_ (methacholine PC_20_) < 16 mg/mL) and/or bronchodilator responsiveness (an increase of FEV_1_% predicted > 12% and FEV_1_ > 200 mL following inhalation of 200 μg salbutamol); 2) age was not less than 18 and smoking history was no more than l0 pack-years; 3) study designs were randomized placebo-controlled trials; 4) intervention treatment was oral CRTH2 antagonists regardless of dose, frequency, and durations; 5) outcomes included but not limited to lung function, asthma control and quality of life scores, sputum and blood eosinophil count, fractional exhaled nitric oxide (FeNO), asthma exacerbations, rescue use of short-acting β_2_ agonists (SABA), and adverse events. Retrospective, observational, cohort or case control studies were excluded.

### Study selection

Two investigators independently performed the study selection in two phases. First, they screened the titles and abstracts of all identified studies to discard duplicated and nonrandomized controlled studies. Then, eligible studies were extracted by reviewing full texts according to the previously defined study inclusion and exclusion criteria. Disagreements were resolved by consensus or consulting a third investigator.

### Data extraction and quality assessment

Two investigators independently and separately conducted the data extraction and quality assessment. Data from eligible studies were extracted in a standard form recommended by Cochrane [15] including authors, publication year, study design, participant characteristics, population, interventions, concomitant treatment, outcome measures and study results. Cochrane risk of bias tool was used to assess the risk of bias in estimating the study outcomes. Each study was assessed for: 1) random sequence generation; 2) allocation concealment; 3) blinding of participants and personnel; 4) blinding of related outcomes assessment; 5) incomplete outcome data; 6) selective reporting; and 7) other biases. For any missing data or information, we contacted corresponding authors by e-mail to request the full original data. Any divergence was resolved by mutual consensus in the presence of a third investigator.

### Statistical analysis

Statistical analysis was accomplished by an independent statistician using Cochrane systematic review software Review Manager (RevMan; Version 5.3.5, the Cochrane Collaboration) and Stata (version 14.0, Stata Corporation, USA). *P* value < 0.05 was defined as statistical significance and the results were showed in forest plots. We conducted a systematic review when data could not be pooled in meta-analysis.

Continuous variables were expressed as mean and standard deviation (SD), while dichotomous variables were shown as frequency and proportion. Mean differences (MD) and 95% confidence interval (CI) were calculated for continuous data, and risk ratio (RR) or risk difference (RD) combined with 95% CI for dichotomous data. If a study presents more than two interventions, they were combined into a single intervention group according to the Cochrane handbook [15]. Heterogeneity was quantified by *I*^*2*^ statistic and chi-squared test with *P* < 0.1 and *I*^*2*^ > 50% indicating significant heterogeneity. Random-effects model was applied in the statistical heterogeneity; otherwise fixed-effects model was used. Publication bias was tested by Funnel plot with Egger’s and Begg’s tests. All analyses were conducted based on the intention-to-treat principle. The potential influence of pre-specified factors, such as types of CRTH2 antagonists, presence of concomitant treatment, treatment duration, asthma severity, on the effect estimates was further explored via random-effects model meta-regression when an outcome of interest was reported by at least three RCTs in each subgroup.

## Results

**Fig. 1:**
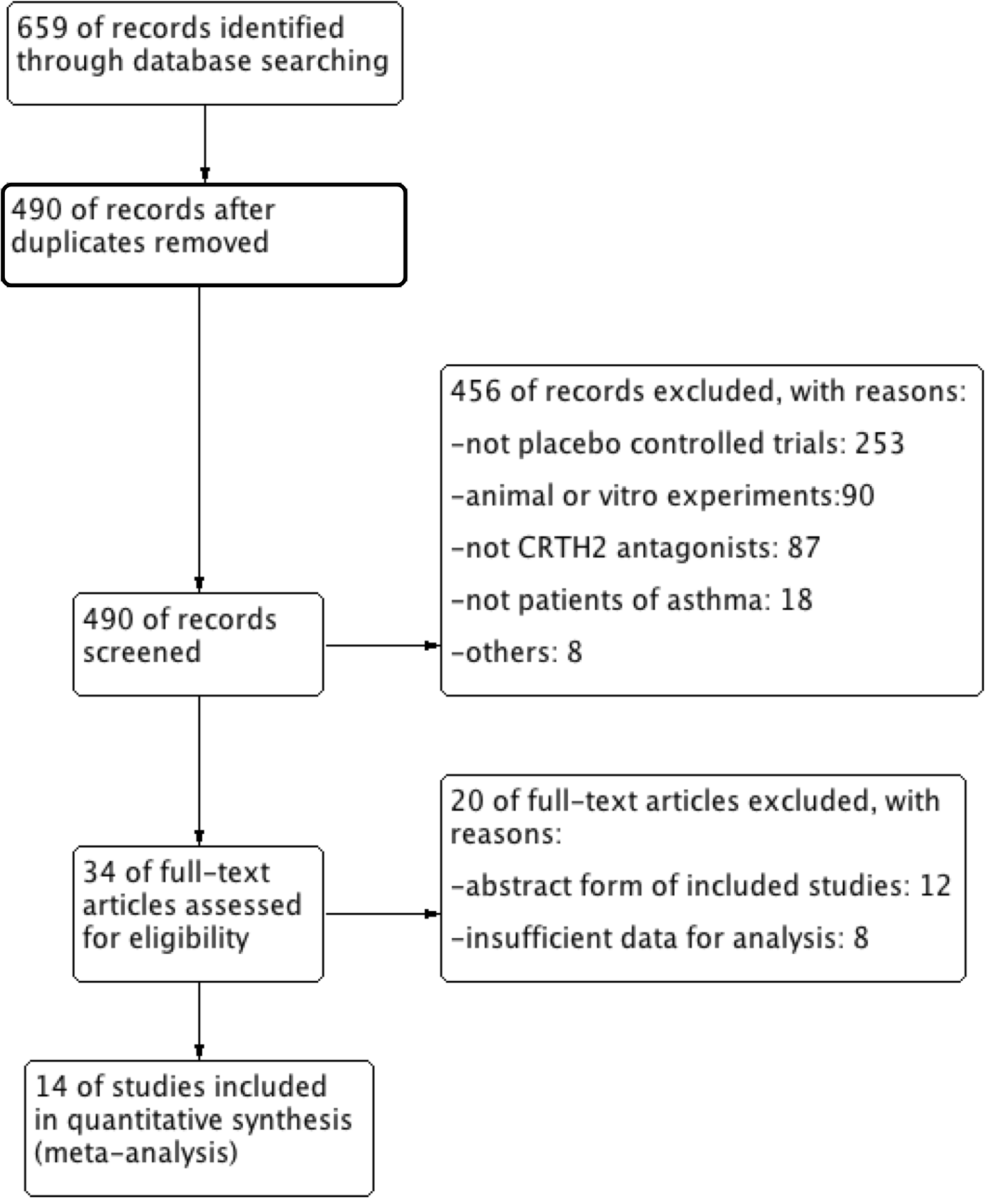
Flow diagram. CRTH2, chemoattractant receptor-homologous molecule expressed on Th2 cells

A total of 659 potentially relevant articles were identified, and 490 articles were screened for eligibility after removal of 169 duplicate records. After reviewing the titles and abstracts, we identified and retrieved 34 studies for later full-text assessment due to the discard of non-RCTs (*n* = 253), animal experiments (*n* = 90), non-CRTH2 antagonists (*n* = 87), non-asthmatic patients (*n* = 18), and others going against our inclusion criteria (*n* = 8). Finally, 14 studies were included for our systematic review and meta-analysis because 20 studies were excluded owing to abstract form of included studies (*n* = 12) and insufficient data for analysis (*n* = 8). (Fig. 1).

### Study characteristics

The characteristics of included RCTs and baseline characteristics of the patients enrolled were summarized in Table 1 and Table 2, respectively. There were eleven [11, 12, 14, 16–23]parallel and three [13, 24, 25] crossover RCTs, and twelve RCTs were designed as multicenter trials [11–14, 16–18, 21–25]. Overall, 4671 participants were included, among which 2581 patients were assigned to receive CRTH2 antagonists, while 2090 patients were administered placebo. Different trials reported inconsistent types of CRTH2 antagonists: 3 for OC000459 [11–13], 3 for Fivipiprant (QAW039) [16, 18, 20], 2 for BI 671800 [14, 25] and AZD1981 [21, 23], and 1 for Setipiprant [24], AMG 853 [17], ARRY-502 [22], and BI 1021958 [19]. Corticosteroids were used as concomitant treatments by all participants in seven trials [14, 16, 17, 20, 21, 23, 25] and SABA was allowed if necessary in all trails. Treatment duration ranged from 5 days to 12 weeks and follow-up varied from 15 days to about 24 weeks.

**Table 1:** Characteristics of randomized controlled trials included

Source	Study Design	Participants characteristics	Population	Intervention (Drug, Dose, Frequency)	Control	Concomitant treatments	Duration	Follow-up	Outcomes^*^
Barnes 2012 [11]	Multi-center, parallel-group, RCT	Steroid-free, moderate persistent asthma	132	OC000459, 200 mg, Twice daily	Placebo	SABA	4 weeks	10 weeks	①⑤⑥⑦⑨⑪⑫⑯⑲
Pettipher 2014 [12]	Multi-center, parallel-group, RCT	Mild-to-moderate persistent steroid-free asthma	482	OC000459, 25 mg, Once daily	Placebo	SABA	12 weeks	20–22 weeks	①⑤⑪⑯⑰⑱
				OC000459, 200 mg, Once daily					
				OC000459, 100 mg, Twice daily					
Singh 2013 [13]	Multi-center, two-period, cross-over, RCT	Steroid-naïve mild allergic asthma	21	OC000459, 200 mg, Twice daily	Placebo	SABA	16 days	35–41 days	①③⑫⑭⑮⑯
Hall (trial 1) 2015 [14]	Multi-center, parallel-group, RCT	Symptomatic, mild-to-moderate, steroid-naïve asthma	317	BI 671800, 50 mg, Twice daily	Placebo	SABA	6 weeks	10 weeks	③⑯⑰⑱⑲
				BI 671800, 200 mg, Twice daily					
				BI 671800, 400 mg, Twice daily					
Hall (trial 2) 2015 [14]		Symptomatic, mild-to-moderate asthmatic patients on ICS	176	BI 671800, 400 mg, Twice daily	Placebo	SABA, ICS		10–12 weeks	③⑯⑰⑱⑲
Bateman 2017 [16]	Multi-center, parallel-group, RCT	Allergic asthma inadequately controlled with low-dose ICS	901	Fevipiprant, 1 mg/3 mg/ 10 mg or 2 mg, Once daily or twice daily	placebo	SABA or ICS	12 weeks	22–24 weeks and 2 days	⑪⑭⑯⑰⑱⑲
				Fevipiprant, 30 mg/50 mg/ 75 mg or 25 mg, Once daily or twice daily					
				Fevipiprant, 150 mg/300 mg or 75 mg/ 150 mg, Once daily or twice daily					
				Fevipiprant, 450 mg, Once daily					
Busse 2013 [17]	Multi-center, parallel-group, RCT	Inadequately controlled, moderate-to-severe asthma	396	AMG 853, 5 mg, Twice daily	Placebo	SABA, ICS	12 weeks	18 weeks	①②③④⑤⑥⑧⑨⑩⑪⑫⑭⑯⑰⑱⑲
				AMG 853, 25 mg, Twice daily					
				AMG 853, 100 mg, Twice daily					
				AMG 853, 200 mg, Twice daily					
Erpenbeck 2016 [18]	Multi-center, parallel-group, RCT	mild-to-moderate persistent allergic asthma	170	Fevipiprant, 500 mg, Twice daily	Placebo	SABA	4 weeks	8 weeks	⑧⑩⑯⑰
Fowler 2017 [19]	Single-center, parallel-group, RCT	Well controlled mild asthma	84	BI 1021958, 5/20/60/200 mg or 40/150/400 mg, Twice daily or once daily	Placebo	SABA	15 days	15 days	⑯⑰⑱⑲
Gonem 2016 [20]	Single-center, parallel-group, RCT	Persistent, moderate-to-severe eosinophilic asthma	61	Fevipiprant, 225 mg, Twice daily	Placebo	SABA, ICS, LABA, or oral prednisone	12 weeks	20 weeks	①②⑧⑨⑫⑬⑭⑯⑰
Kuna (trial 1) 2016 [21]	Multi-center, parallel-group, RCT	Stable asthma withdrawn from ICS	113	AZD1981, 1000 mg, Twice daily	Placebo	SABA	4 weeks	8 weeks	①②⑤⑦⑥⑩⑫⑬⑭⑯⑰⑱⑲
Kuna (trial 2) 2016 [21]	Multi-center, parallel-group, RCT	Uncontrolled moderate-to-severe asthma despite moderate-to-high dose of ICS	368	AZD1981, 50 mg, Twice daily	Placebo	SABA, ICS	4 weeks	8 weeks	①⑦⑯⑰⑱⑲
				AZD1981, 400 mg, Twice daily					
				AZD1981, 1000 mg, Twice daily					
Wenzel 2014 [22]	Multi-center, parallel-group, RCT	Steroid-free, mild atopic asthma	184	ARRY-502, 200 mg, Twice daily	Placebo	SABA	4 weeks	6 weeks	①⑧⑨⑪⑯⑲
Bateman 2018 [23]	Multi-center, parallel-group, RCT	Persistent allergic asthma	1144	AZD1981, 80/200 mg, once daily, or 10/40/100 mg, twice daily	Placebo	ICS, LABA, SABA	12 weeks	15 weeks	①⑧⑪⑯⑰⑱
Diamant 2014 [24]	Multi-center, two-period, cross-over, RCT	Stable, allergic asthma	14	Setipiprant, 1000 mg, Twice daily	Placebo	SABA	5 days	37 days	⑬⑭⑮
Miller 2017 [25]	Multi-center, three-period, cross-over, RCT	Mild-to-moderate symptomatic asthma	108	BI 671800, 400 mg, Once daily (a.m)	Placebo	SABA, ICS	12 weeks	16–18 weeks	③⑧⑯⑰⑱⑲

^*^Outcomes include: ① change of pre-bronchodilator FEV_1_, ② change of post-bronchodilator FEV_1_, ③ change of pre-bronchodilator FEV_1_% predicted, ④ change of post-bronchodilator FEV_1_% predicted, ⑤ change of morning PEF, ⑥ change of evening PEF, ⑦ change of FVC, ⑧ change of ACQ scores, ⑨ change of AQLQ scores, ⑩ change of SABA use, ⑪ incidence of asthma exacerbation, ⑫ change of sputum eosinophils, ⑬ change of blood eosinophils, ⑭ change of FeNO, ⑮ change of methacholine PC_20_, ⑯ incidence of adverse events, ⑰ incidence of severe adverse events, ⑱ incidence of treatment related adverse events, ⑲ incidence of adverse events leading to treatment withdrawal

*ACQ* asthma control questionnaire, *AQLQ* asthma quality of life questionnaire, *FeNO* fractional exhaled nitric oxide, *FEV*_*1*_ forced expiratory volume in one second, *FVC* forced vital capacity, *Methacholine PC*_*20*_ the provocation concentration of methacholine causing a 20% fall in FEV_1_, *NM* not mentioned, *PEF* peak expiratory flow, *RCT* randomized controlled trial, *SABA* short-acting beta-agonists

**Table 2:** Baseline characteristics of patients in each enrolled trial

Source	No.	Age (years)^*^	Female (%)	BMI (kg/m^2^)^*^	Smoking (Pack-year)	Pre-bronchodilator FEV_1_% predicted^*^	FeNO (ppb)^*^	Positive atopic status (%)	Diagnosis or duration of asthma (years)^*^
Barnes 2012 [11]	65	43.4 (18–55)^**^	41.53	NM	≤10	NM	NM	100	6.5 (6.29)
Pettipher 2014 [12]	125	40.4 (11.4)	70	NM	< 10	71.5 (6.1)	NM	NM	NM
	123	39.7 (10.2)	81	NM	< 10	71.0 (6.2)	NM	NM	NM
	117	38.9 (11.4)	76	NM	< 10	71.5 (6.9)	NM	NM	NM
Singh 2013 [13]	21	31.1 (7.1)	14.3	NM	< 10	87.4 (12.0)	32.7 (21.8)	100	NM
Hall (trial 1) 2015 [14]	77	39.1 (11.5)	53.2	27.0 (4.6)	< 10	71.4 (7.3)	NM	75.3	NM
	83	35.1 (11.1)	50.6	25.9 (4.3)	< 10	73.3 (7.3)	NM	78.3	NM
	79	37.5 (12.2)	54.4	26.5 (4.7)	< 10	73.6 (6.9)	NM	84.8	NM
Hall (trial 2) 2015 [14]	81	41.8 (12.7)	61.7	27.6 (4.1)	< 10	72.6 (7.6)	NM	82.7	NM
Bateman 2017 [16]	201	45.2 (12.1)	59.2	28.1 (5.7)	0	64.4 (9.6)	NM	100	20.5 (14.9)
	219	45.6 (12.1)	54.34	26.9 (5.0)	0	64.1 (10.1)	NM	100	20.5 (14.9)
	212	43.5 (12.3)	60.85	27.4 (5.5)	0	64.5 (9.7)	NM	100	18.4 (14.0)
	133	45.8 (12.5)	54.89	27.8 (5.0)	0	63.7 (10.5)	NM	100	21.2 (15.0)
Busse 2013 [17]	79	44.7 (11.5)	65.8	29.6 (6.2)	< 10	68.2 (7.9)	31.9 (21.4)	93.7	28.0 (14.1)
	79	45.0 (11.3)	58.2	32.0 (6.5)	< 10	67.1 (7.9)	30.9 (30.5)	91.1	24.8 (13.2)
	79	44.6 (11.4)	69.8	31.9 (8.0)	< 10	66.7 (8.5)	28.3 (23.2)	91.1	28.9 (14.5)
	80	43.7 (11.4)	40.0	31.4 (7.2)	< 10	66.1 (8.9)	33.5 (31.6)	95.0	27.3 (12.8)
Erpenbeck 2016 [18]	82	41 (12.9)	24	28.5 (5.81)	< 10	71.5 (7.11)	32 (NM)	100	NM
Fowler 2017 [19]	63	33.1 (10.9)	NM	24.2 (2.9)	< 10	85.2 (15.0)	NM	NM	NM
Gonem 2016 [20]	30	50 (17)	40	31.0 (5.9)	NM	72.5 (23.8)	30 (24)	87	32 (16)
Kuna (trial 1) 2016 [21]	57	38.4 (NM)	16	26.3 (NM)	< 10	82.6 (NM)	NM	100	13 (NM)
Kuna (trial 2)2016 [21]	95	43.3 (NM)	28	26.9 (NM)	< 10	66.2 (NM)	NM	72	11.1 (NM)
	90	43.0 (NM)	21	27.0 (NM)	< 10	68.5 (NM)	NM	77	12.1 (NM)
	92	43.5 (NM)	37	27.2 (NM)	< 10	69.0 (NM)	NM	64	10 (NM)
Wenzel 2014 [22]	93	35 (18–68)^**^	49	26.0 (19.3–34.6)^**^	< 10	73.4 (60–84)^***^	47.5 (26–244)^**^	100	NM
Bateman 2018 [23]	976	41.0 (NM)	49.5	27.5 (NM)	≤10	NM	NM	100	18.2 (NM)
Diamant 2014 [24]	18	30.6 (21–46)^**^	0	25.58 (NM)	< 10	NM	51.6 (38.5)	100	NM
Miller 2017 [25]	108	41.1 (12.4)	53.7	28.8 (4.8)	< 10	72.8 (7.6)	NM	63.9	28.2 (12.9)

Data was expressed as ^*^mean (SD), ^**^ mean (range), ^***^ median (range)

*BMI* body mass index, *FeNO* fractional exhaled nitric oxide, *FEV*_*1*_ forced expiratory volume in one second, *NM* not mentioned

The mean age of the participants ranged from 33.1 to 50 years old, and the mean FEV_1_% predicted values at baseline was between 64.2 and 85.2%. Body mass index (BMI) was reported to be from 24.2 to 32.0 kg/m^2^ in 11 studies [14, 16–25], and FeNO varied from 30.0 to 51.6 ppb in 5 studies [13, 17, 18, 20, 24]. All participants were non-smokers or ex-smokers with a smoking history ≤10 pack-years. One study [24] only included male participants, and eight studies [11, 13, 16, 18, 21–24] enrolled allergic asthmatics. Four studies [17, 20, 21, 23] involved patients with moderate-to-severe asthma, eight studies [11–14, 18, 19, 22, 25] included patients with mild to moderate asthma, and the remaining three studies [16, 21, 24] did not specify asthma severity.

### Quality assessment

**Fig. 2:**
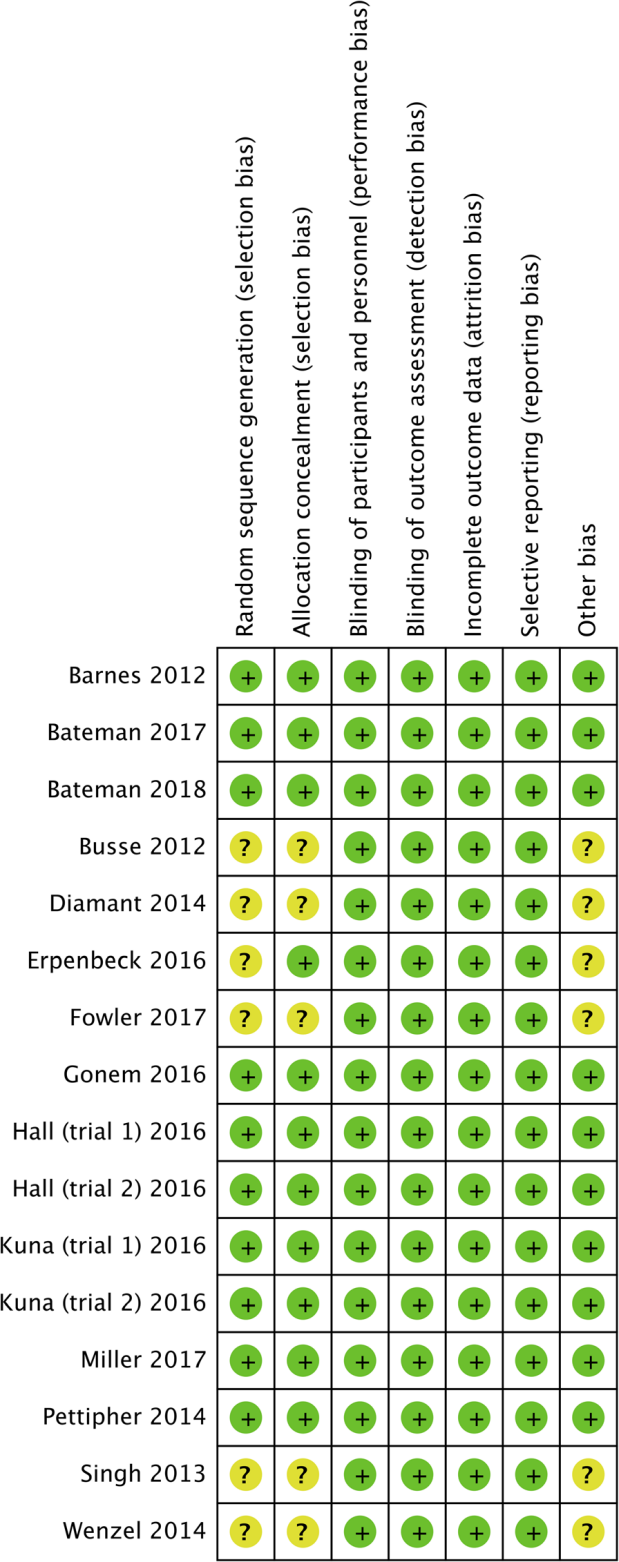
Risk of bias summary

Based on the six domains, all the included studies showed low risk of bias (Fig. 2). The method used in randomization sequence generation and allocation concealment was clearly described in all the studies except seven studies [13, 17–19, 22–24]. All the 13 studies were double-blinded and reported complete outcome data.

### Outcomes

#### FEV_1_

Ten studies [11–14, 17, 20–23, 25] examined the effect of CRTH2 antagonists compared with placebo on FEV_1_, of which eight studies [11–13, 17, 20–23] reported FEV_1_ in liters (L) and four [13, 14, 17, 25] in FEV_1_% predicted. In terms of pre- and post-bronchodilator FEV_1_, eight studies [11–13, 17, 20–23] and four studies [13, 14, 17, 24] showed pre-bronchodilator FEV_1_ (L) and FEV_1_% predicted, while three studies [17, 20, 21] and one study [17] evaluated post-bronchodilator FEV_1_ (L) and FEV_1_% predicted, respectively. The mean difference in pre-bronchodilator FEV_1_ (L) from baseline was computed for five studies [11–13, 21, 22] of no corticosteroids use and four studies [17, 20, 21, 23] of corticosteroids use.

**Fig. 3:**
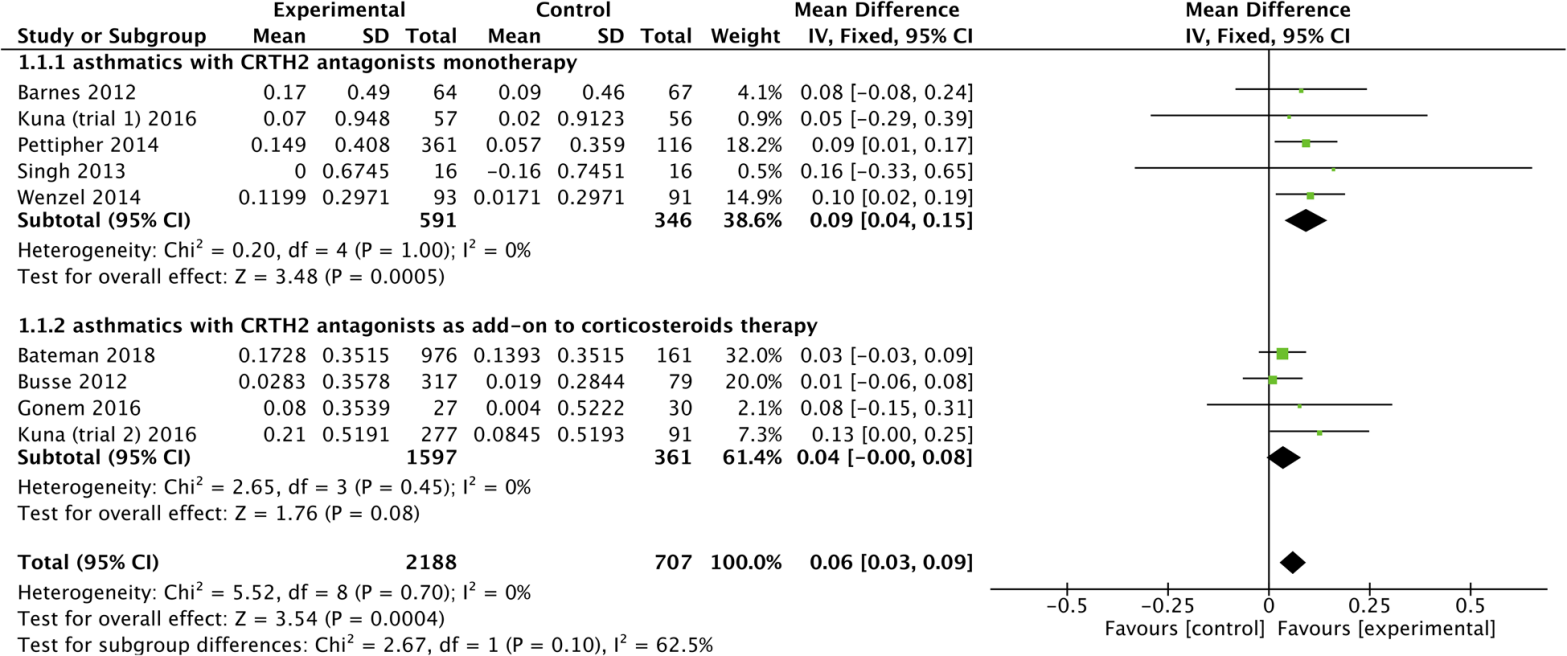
The effect of CRTH2 antagonists vs placebo on FEV_1._ CI, confidential interval; CRTH2, chemoattractant receptor-homologous molecule expressed on Th2 cells; FEV_1_, forced expiratory volume in one second; SD, standard deviation; vs, versus

No statistical heterogeneity (*I*^*2*^ = 0%, *P* = 0.70) (Fig. 3) or publication bias (Begg’s test = 0.754, Egger’s test = 0.307) (Additional file 1: Figure S1) were detected in the assessment of pre-bronchodilator FEV_1_ (L). Compared with placebo, CRTH2 antagonists significantly improved pre-bronchodilator FEV_1_ (L) (MD = 0.06, 95% CI 0.03 to 0.09, *P* = 0.0004). Meta-regression indicated that the pooled effect of pre-bronchodilator FEV_1_ (L) was associated with neither treatment duration (*P* = 0.994) (Additional file 1: Figure S2), asthma severity (*P* = 0.150) (Additional file 1: Figure S3), nor concomitant treatment (*P* = 0.146) (Additional file 1: Figure S4). The limited number of studies precluded further assessment of the impacts of different CRTH2 types on pre-bronchodilator FEV_1_ (L), but we could separately extract patients treated with CRTH2 antagonists alone or CRTH2 antagonist combined with corticosteroids. In such a subgroup analysis, we found CRTH2 antagonists monotherapy, instead of CRTH2 antagonists as an add-on treatment to corticosteroids, significantly improved pre-bronchodilator FEV_1_ (L) (MD = 0.09, 95% CI, 0.04 to 0.15, *P* = 0.0005) (Fig. 3).

Similarly, pre-bronchodilator FEV_1_% predicted could be significantly improved in asthmatics with CRTH2 antagonists monotherapy (MD = 3.65, 95% CI 1.15 to 6.14, *P* = 0.004) rather than CRTH2 antagonists as add-on treatment to corticosteroids therapy (MD = 1.03, 95% CI -0.83 to 2.90, *P* = 0.28), however, moderate statistical heterogeneity (*I*^*2*^ = 63%, *P* = 0.07) was found in combination therapy of CRTH2 antagonists and corticosteroids but not in CRTH2 antagonists alone (*I*^*2*^ = 0%, *P* = 0.82). In the pooled analysis, we found no superior effect of CRTH2 antagonists compared to the placebo on pre-bronchodilator FEV_1_% predicted (MD = 1.75, 95% CI -0.04 to 3.53, *P* = 0.06), but it also showed moderate statistical heterogeneity (*I*^*2*^ = 63%, *P* = 0.03) (Additional file 1: Figure S5).

As for the post-bronchodilator FEV_1_ (L) and FEV_1_% predicted, no effect of CRTH2 antagonists was observed neither in the pooled (FEV_1_ (L): MD = 0.05, 95% CI -0.07 to 0.17, *P* = 0.44) nor subgroup analysis of CRTH2 antagonists as add-on treatment to corticosteroids therapy (FEV_1_ (L): MD = 0.06, 95% CI -0.09 to 0.22, *P* = 0.42; FEV_1_% predicted: MD = − 0.09, 95% CI -1.65 to 1.47, *P* = 0.91), but moderate-to-high statistical heterogeneity of FEV_1_ (L) was found in asthmatics with CRTH2 antagonists as add-on treatment to corticosteroids (*I*^*2*^ = 75%, *P* = 0.04) and pooled asthmatics (*I*^*2*^ = 52%, *P* = 0.13) (Additional file 1: Figure S6).

### Forced vital capacity (FVC)

Two studies with three trials [11, 21] reported the effect of CRTH2 antagonists compared to placebo on FVC, of which two trials [11, 21] administered CRTH2 antagonists alone. FVC could not be significantly improved by either overall CRTH2 antagonists (MD = 0.03, 95% CI -0.12 to 0.19, *P* = 0.67) or CRTH2 antagonist monotherapy (MD = 0.05, 95% CI -0.14 to 0.24, *P* = 0.61) even though no statistical heterogeneities were detected (*I*^*2*^ = 0%, *P* = 0.79) (Additional file 1: Figure S7). One trial [21] used CRTH2 antagonist as add-on treatment to corticosteroids, but the result showed no improvement in FVC.

### Peak expiratory flow (PEF)

**Fig. 4:**
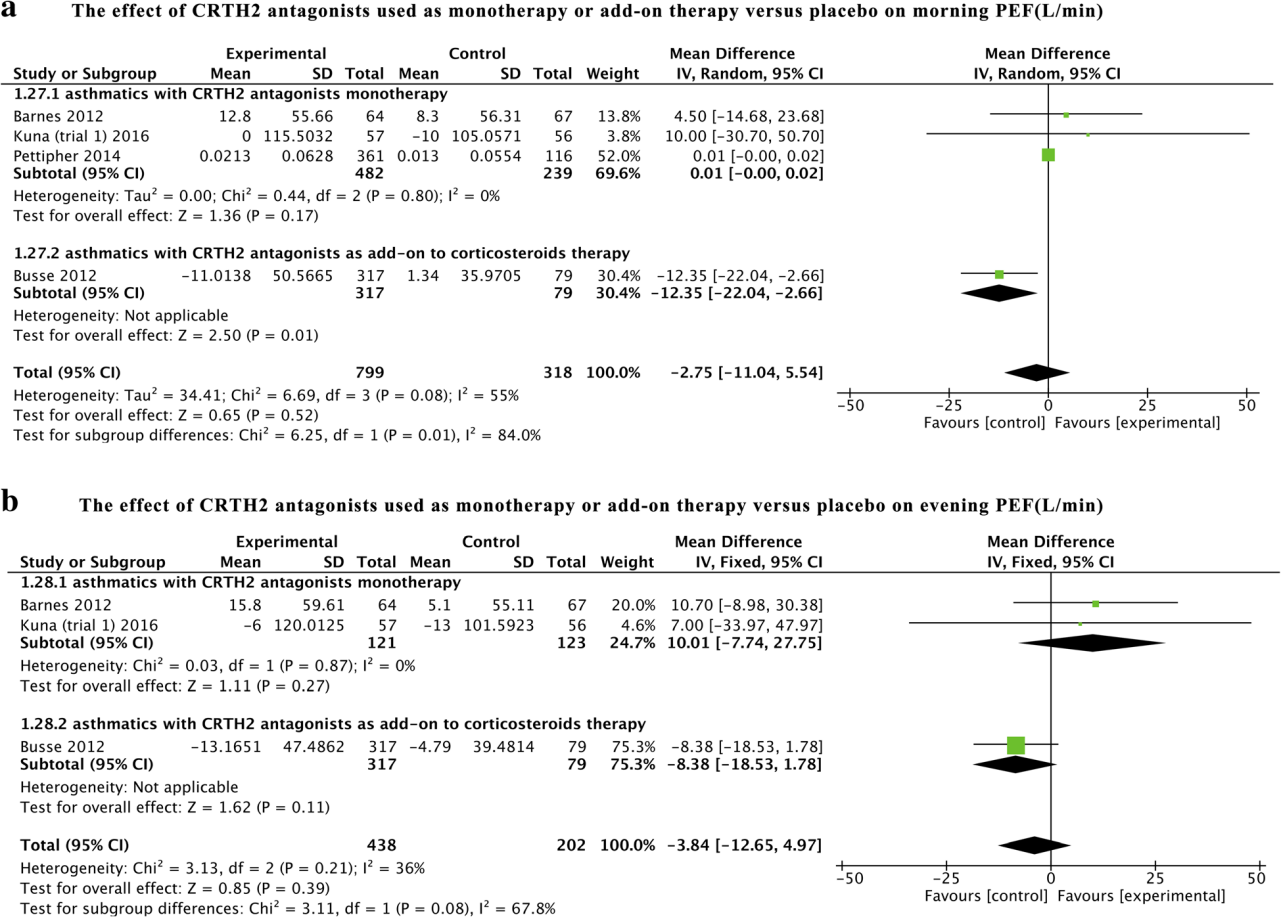
The effect of CRTH2 antagonists vs placebo on PEF. CI, confidential interval; CRTH2, chemoattractant receptor-homologous molecule expressed on Th2 cells; PEF, peak expiratory flow; SD, standard deviation; vs, versus

PEF was reported as morning and evening PEF in four [12, 13, 17, 21] and three [11, 17, 21] studies, respectively. Three studies [11, 12, 21] assessed the effect of CRTH2 antagonists monotherapy, while one study [17] also showed CRTH2 antagonists as add-on treatment. No statistical heterogeneities were found except for that in morning PEF in the pooled analysis (*I*^*2*^ = 55%, *P* = 0.08) (Fig. 4). No significant improvements of morning and evening PEF were shown in asthmatics with CRTH2 antagonists monotherapy (morning PEF: MD = 0.01, 95% CI -0.00 to 0.02, *P* = 0.17; evening PEF: MD = 10.01, 95% CI -7.74 to 27.75, *P* = 0.27) or in pooled analysis (morning PEF: MD = − 2.75, 95% CI -11.04 to 5.54, *P* = 0.52; evening PEF: MD = − 3.84, 95% CI -12.65 to 4.97, *P* = 0.85). However, subgroup analysis indicated that CRTH2 antagonists as add-on treatment to corticosteroid could reduce morning PEF (MD = − 12.35, 95% CI -22.04 to − 2.66, *P* = 0.01) instead of evening PEF (MD = − 8.38, 95% CI -18.53 to 1.78, *P* = 0.11).

### Asthma control questionnaire (ACQ)

**Fig. 5:**
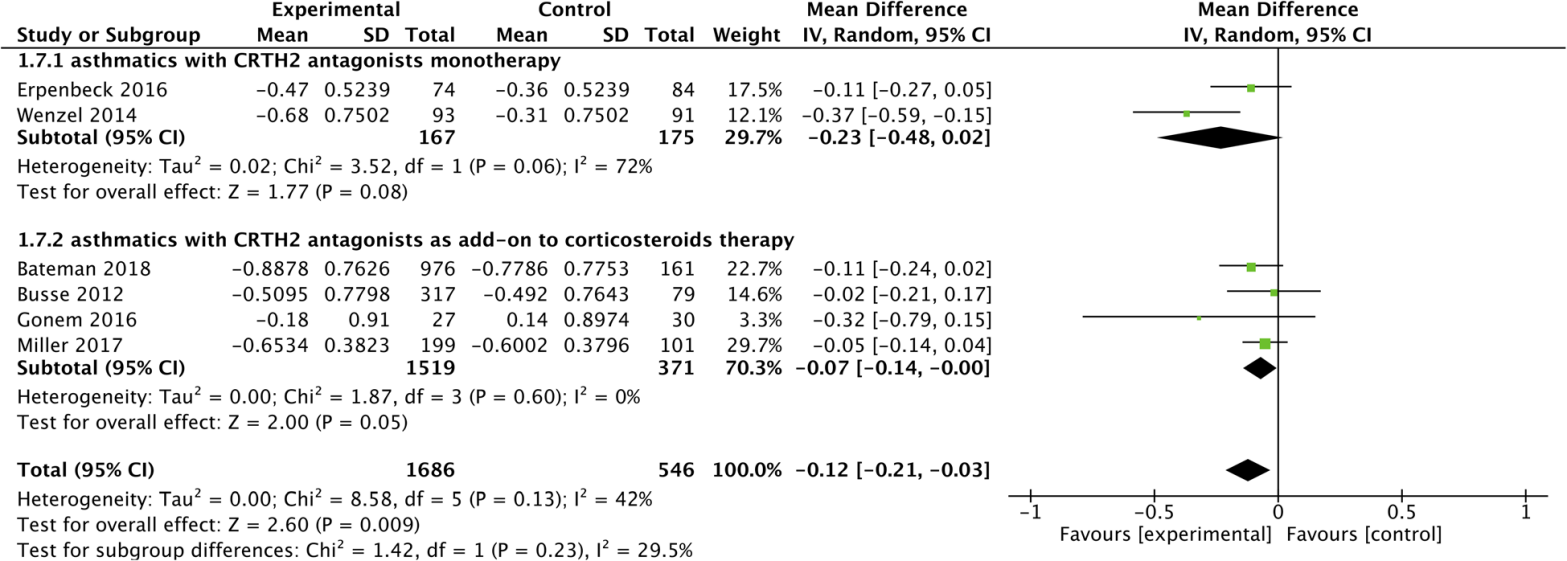
The effect of CRTH2 antagonists vs placebo on ACQ. ACQ, asthma control questionnaire; CI, confidential interval; CRTH2, chemoattractant receptor-homologous molecule expressed on Th2 cells; SD, standard deviation; vs, versus

The effect of CRTH2 antagonists on ACQ scores was reported in six studies [17, 18, 20, 22, 23, 25], of which four [17, 20, 23, 25] in asthmatics with CRTH2 antagonists as add-on treatment and two [18, 22] with CRTH2 antagonists monotherapy. Moderate statistical heterogeneity was noticed in CRTH2 antagonists monotherapy (*I*^*2*^ = 72%, *P* = 0.06) , and the results showed that CRTH2 antagonists in general could significantly reduce ACQ score in asthmatics (MD = − 0.12, 95% CI -0.21 to − 0.03, *P* = 0.009), but little effect was observed in asthmatics with CRTH2 antagonists used as neither monotherapy (MD = − 0.23, 95% CI -0.48 to 0.02, *P* = 0.08) nor add-on treatment to corticosteroids (MD = − 0.07, 95% CI -0.14 to 0.001, *P* = 0.045) (Fig. 5).

### Asthma quality of life questionnaire (AQLQ)

**Fig. 6:**
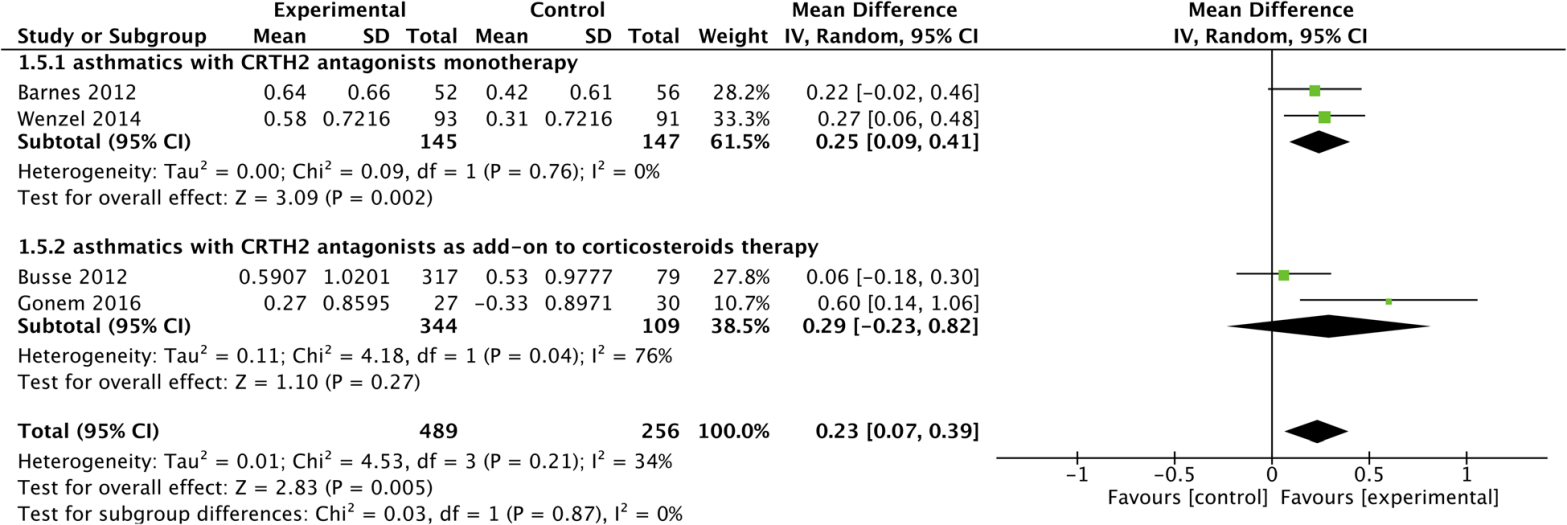
The effect of CRTH2 antagonists vs placebo on AQLQ. AQLQ, asthma quality of life questionnaire; CI, confidential interval; CRTH2, chemoattractant receptor-homologous molecule expressed on Th2 cells; SD, standard deviation; vs, versus

Four studies [11, 17, 20, 22] reported AQLQ score in asthmatic patients with treatment of CRTH2 antagonists, and we did not find statistical heterogeneities except for CRTH2 antagonists used as add-on treatment to corticosteroids (*I*^*2*^ = 76%, *P* = 0.04). CRTH2 antagonists were shown to significantly improve AQLQ score compared to placebo (MD = 0.23, 95% CI 0.07 to 0.39, *P* = 0.005), however, in the subgroup analysis, it resulted in significant improvement of AQLQ score in patients with CRTH2 antagonists monotherapy (MD = 0.25, 95% CI 0.09 to 0.41, *P* = 0.002) rather than CRTH2 antagonists as add-on treatment (MD = 0.29, 95% CI -0.23 to 0.82, *P* = 0.27) (Fig. 6).

### Rescue use of SABA

**Fig. 7:**
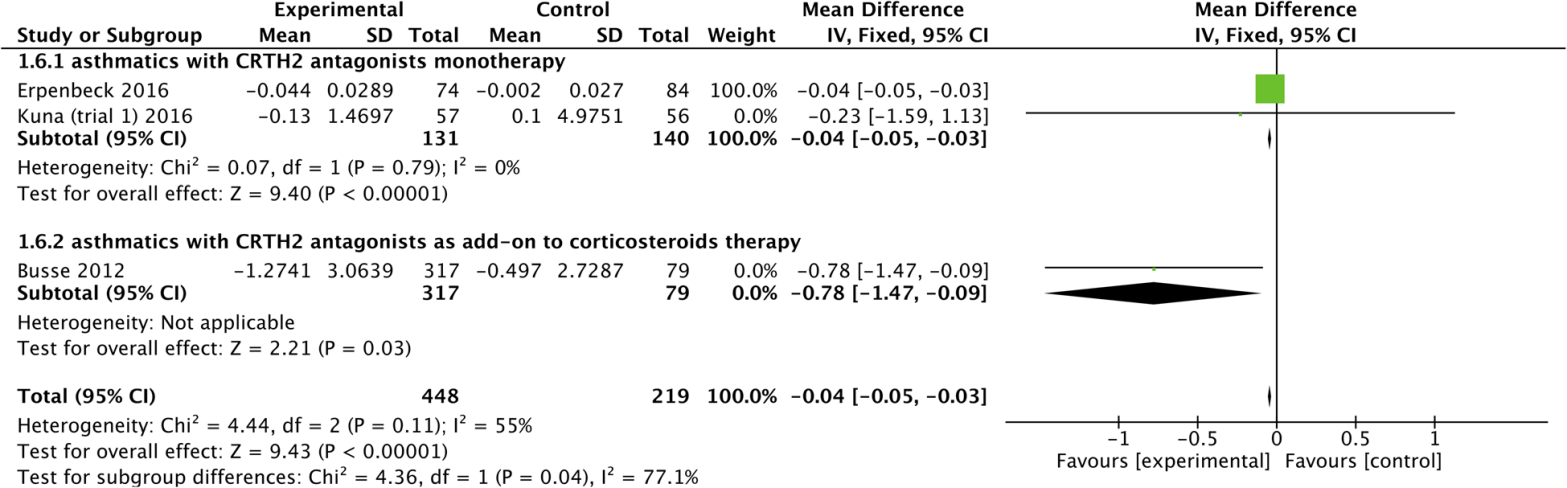
The effect of CRTH2 antagonists vs placebo on SABA use. CI, confidential interval; CRTH2, chemoattractant receptor-homologous molecule expressed on Th2 cells; SABA, short-acting β_2_ agonists; SD, standard deviation; vs, versus

Three studies [17, 18, 21] reported the effect of CRTH2 antagonists on the rescue use of SABA, and we found that CRTH2 antagonists significantly reduced SABA usage (MD = − 0.04, 95% CI -0.05 to − 0.03, *P* < 0.00001), regardless of monotherapy (MD = − 0.04, 95% CI -0.05 to − 0.03, *P* < 0.00001) or serving as an add-on therapy to corticosteroids (MD = − 0.78, 95% CI -1.47 to − 0.09, *P* = 0.03) (Fig. 7).

### Asthma exacerbations

**Fig. 8:**
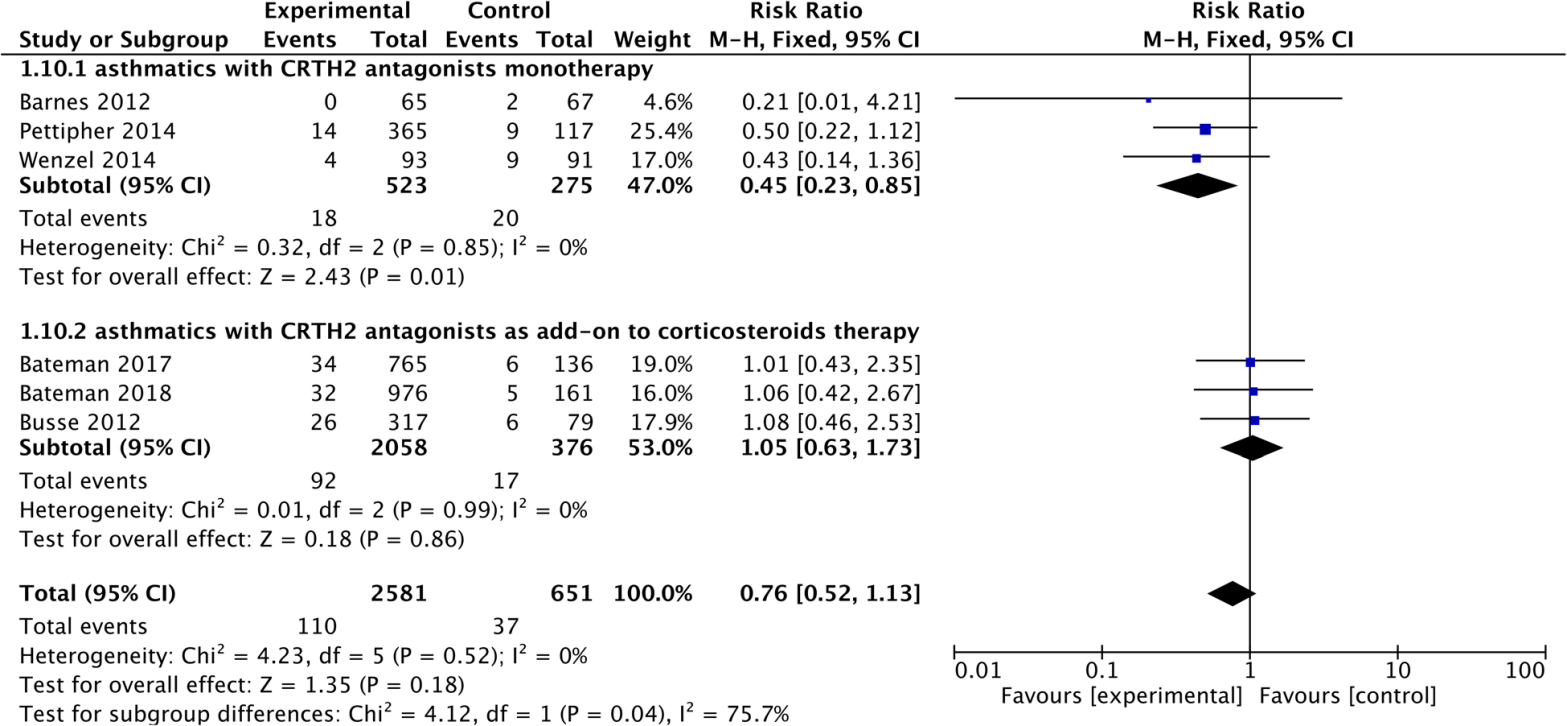
The effect of CRTH2 antagonists on asthma exacerbations. CI, confidential interval; CRTH2, chemoattractant receptor-homologous molecule expressed on Th2 cells; vs, versus

Six studies [11, 12, 16, 17, 22, 23] presented data on asthma exacerbations, and they all included patients with exacerbations based on a decline of more than 30% from the baseline in morning PEF on two or more consecutive mornings, or a worsening of asthma symptoms requiring treatment with systemic corticosteroids or increased doses of rescue medication, and/or the need for asthma-related hospitalization/emergency room visit. The pooled analysis showed no significant difference in the incidence of asthma exacerbations (RR = 0.76, 95% CI 0.52 to 1.13, *P* = 0.18) between asthmatics treated with CRTH2 antagonists and placebo, however, in the subgroup analysis, we found asthma exacerbations were significantly reduced in CRTH2 monotherapy (RR = 0.45, 95% CI 0.23 to 0.85; *P* = 0.01) rather than CRTH2 antagonists as add-on treatment (RR = 1.05, 95% CI 0.63 to 1.75, *P* = 0.86) (Fig. 8). No statistical heterogeneities were detected in pooled (*I*^*2*^ = 0, *P* = 0.52) and subgroup (monotherapy: *I*^*2*^ = 0, *P* = 0.85; add-on treatment: *I*^*2*^ = 0, *P* = 0.99) analysis.

### Adverse events

**Fig. 9:**
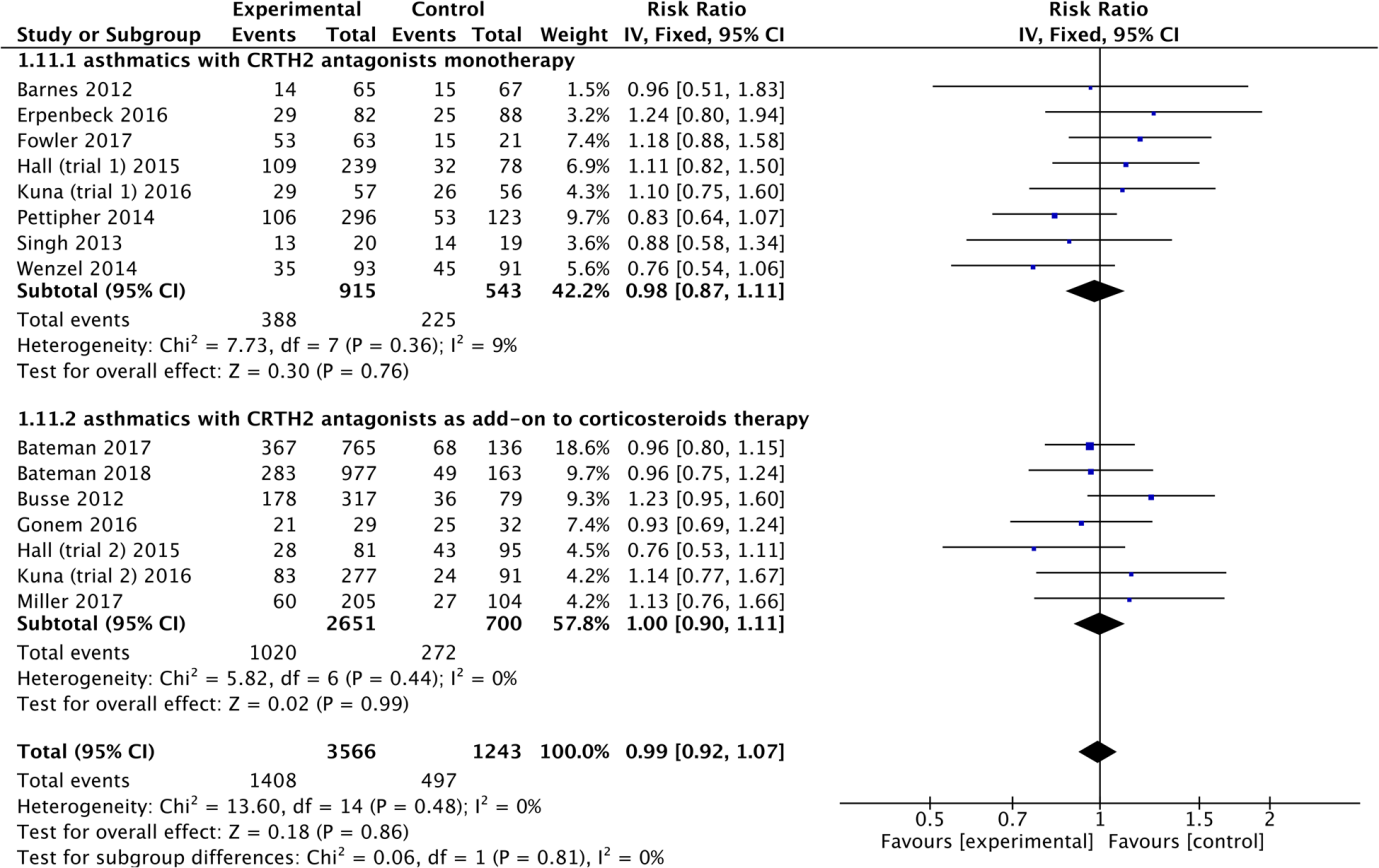
The effect of CRTH2 antagonists vs placebo on adverse events. CI, confidential interval; CRTH2, chemoattractant receptor-homologous molecule expressed on Th2 cells; vs, versus

Adverse events were reported in thirteen studies [11–14, 16–23, 25], of which ten [12, 14, 16–21, 23, 25], eight [12, 14, 16, 17, 19, 21, 23, 25] and eight [11, 14, 16, 17, 19, 21, 22, 25] studies further examined severe adverse events, treatment related adverse events, and adverse events leading to treatment withdrawal, respectively. The most commonly reported adverse events were nasopharyngitis, headache, asthma, infections and gastrointestinal disorders. Each type of adverse events was pooled into our meta-analysis, and no significant statistical heterogeneities were found either in overall or subgroup analysis (Fig. 9 and Additional file 1: Figures S8–S10). In terms of adverse events, severe adverse events and treatment related adverse events, we found similar incidence between CRTH2 antagonists and placebo in pooled analysis (adverse events: RR = 0.99, 95% CI 0.92 to 1.07, *P* = 0.86; severe adverse events: RD = − 0.00, 95% CI -0.01 to 0.00, *P* = 0.43; treatment related adverse events: RR = 1.01, 95% CI 0.75 to 1.34, *P* = 0.97) as well as CRTH2 antagonists monotherapy (adverse events: RR = 0.98, 95% CI 0.87 to 1.11, *P* = 0.76; severe adverse events: RD = − 0.01, 95% CI -0.02 to 0.01, *P* = 0.23; treatment related adverse events: RR = 0.89, 95% CI 0.63 to 1.25, *P* = 0.49) and add-on treatment to corticosteroids (adverse events: RR = 1.00, 95% CI 0.90 to 1.11, *P* = 0.99; severe adverse events: RD = − 0.00, 95% CI -0.01 to 0.01, *P* = 0.93; treatment related adverse events: RR = 1.16, 95% CI 0.77 to 1.74, *P* = 0.48) (Fig. 9 and Additional file 1: Figure S8 and S9). However, significantly lower incidence of adverse events leading to treatment withdrawal was found in CRTH2 antagonists treatment compared to placebo (RD = − 0.02, 95% CI -0.04 to − 0.00; *P* = 0.03), and subgroup analysis showed significantly lesser adverse events leading to treatment withdrawal in CRTH2 antagonists monotherapy (RD = − 0.04, 95% CI -0.08 to − 0.01; *P* = 0.02) rather than CRTH2 antagonists as add-on treatment (RD = − 0.01, 95% CI -0.04 to 0.01; *P* = 0.29) (Additional file 1: Figure S10). Further analysis also demonstrated no evidence of publication bias (Egger’s test = 0.758, Begg’s test = 0.767) (Additional file 1: Figure S11) and no association either between treatment duration and the incidence of adverse events (*P* = 0.139) (Additional file 1: Figure S12) or between concomitant therapy (*P* = 0.827) (Additional file 1: Figure S13) or asthma severity (*P* = 0.415) (Additional file 1: Figure S14) and the incidence of adverse events.

### Sputum and blood eosinophils

Five studies [11, 13, 17, 20, 21] presented data on sputum eosinophils in patients with CRTH2 antagonists treatment, and three studies [20, 21, 24] reported blood eosinophils. However, we could not conduct individual synthesized analysis of each outcome due to the inconsistently reported data, in which eosinophils levels were presented either as amount per gram or percent of the whole white cells, and we were unable to extract mean change of eosinophils after treatment from only mean (range) or geometric mean (95% CI).

Table 3 summarized available studies with sputum or blood eosinophils. Ambiguous results were noticed in sputum eosinophils as some studies [13, 20] showed significant reduction of sputum eosinophils in patients with CRTH2 antagonists treatment compared to placebo while some studies [11, 17, 21] did not find any significant differences. As for blood eosinophils, all three available studies showed that CRTH2 antagonists treatment could not significantly reduce blood eosinophils compared to placebo regardless of being as monotherapy or an add-on treatment to corticosteroids.

**Table 3:** Results of sputum and blood eosinophils, FeNO and Methacholine PC_20_

Source	Groups	Baseline	Treatment endpoint	Change after treatment	*P* value (Intervention vs Placebo)	Significant Difference
*Sputum eosinophils (10* ^*6*^ */g)*						
Singh 2013 [13]	Intervention	NM	0.4^##^	NM	0.002	Yes
	Control	NM	0.75^##^	NM		
Kuna (trial 1) 2016 [21]	Intervention	0.024 (0.00 to 0.53)^§^	0.004 (0.00 to 0.53)^§^	NM	NM	No
	Control	0.033 (0.00 to 1.21)^§^	0.014 (0.00 to 0.73)^§^	NM		
*Sputum eosinophils (%)*						
Barnes 2012 [11]	Intervention	2.1 (NM)^‡^	0.7 (NM)^‡^	3.1 (1.1, 8.8)^§§^	0.37	No
	Control	1.8(NM)^‡^	1.2 (NM)^‡^	1.5 (0.4, 5.3)^§§^		
Singh 2013 [13]	Intervention	6.0 (1.5, 23.9)^‡^	18.1 (10.0, 33.2)^‡^	NM	0.002	Yes
	Control	6.0 (1.5, 23.9)^‡^	5.6 (2.7, 11.6)^‡^			
Busse 2013 [17]	Intervention	2.0 (0 to 93)^§^	NM	−3.5 (−93 to 5)^§^	NM	No
		1.0 (0 to 69)^§^	NM	−0.5 (−68 to 36)^§^	NM	
		1.0 (0 to 91)^§^	NM	0.0 (−72 to 6)^§^	NM	
		1.0 (0 to 80)^§^	NM	0.0 (−24 to 4)^§^	NM	
	Control	0.0 (0 to 53)^§^	NM	2.0 (−2.3 to 84)^§^	–	
Gonem 2016 [20]	Intervention	5.4 (3.1, 9.6)^‡^	1.1 (0.7, 1.9)^‡^	0.22 (0.13, 0.39)^§§^	0.0014	Yes
	Control	4.6 (2.5–8.7)^‡^	3.9 (2.3–6.7)^‡^	0.78 (0.45, 1.33)^§§^		
*Blood eosinophils (10* ^*9*^ */L)*						
Gonem 2016 [20]	Intervention	0.29 (95.03)^#^	0.29 (0.23–0.36)^‡^	1.01 (0.79, 1.28)^§§^	0.44	No
	Control	0.28 (80.63)^#^	0.32 (0.25, 0.41)^‡^	1.13 (0.89, 1.43)^§§^		
Kuna (trial 1) 2016 [21]	Intervention	NM	NM	NM	NM	No
	Control	NM	NM	NM		
Diamant 2014 [24]	Intervention	NM	NM	NM	NM	No
	Control	NM	NM	NM		
*FeNO (ppb)*						
Singh 2013 [13]	Intervention	33.9 (22.4)^*^	26.3 (23.7)^*^	NM	NM	No
	Control	39.3 (23.9)^*^	23.3 (22.8)^*^	NM		
Bateman 2017 [16]	Intervention	NM	NM	NM	> 0.05	No
		NM	NM	NM	> 0.05	
		NM	NM	NM	> 0.05	
	Control	NM	NM	NM	–	
Busse 2013 [17]	Intervention	31.9 (21.4)^*^	NM	0.221	> 0.05	No
		30.9 (30.5)^*^	NM	2.368	> 0.05	
		28.3 (23.2)^*^	NM	−0.080	> 0.05	
		33.5 (31.6)^*^	NM	1.333	> 0.05	
	Control	28.1 (22.0)^*^	NM	−7.000	–	
Gonem 2016 [20]	Intervention	37.72 (4.75)^†^	34.88 (3.97)^†^	−5.82 (−13.79, 2.16)^**^	0.49	No
	Control	43.67 (6.97)^†^	38.48 (4.32)^†^	−2.21 (−10.90, 6.48)^**^		
Kuna (trial 1) 2016 [21]	Intervention	NM	NM	NM	NM	No
	Control	NM	NM	NM		
Diamant 2014 [24]	Intervention	51.6 (38.5)^*^	NM	NM	NM	No
	Control	71.0 (36.5)^*^	NM	NM		
*Methacholine PC*_*20*_ *(mg/mL)*						
Singh 2013 [13]	Intervention	1.48 (3.2)^*^	0.31^*^	NM	NM	No
	Control	1.48 (3.2)^*^	0.39^*^			
Diamant 2014 [24]	Intervention	0.91 (2.21)^††^	0.97 (1.98)^††^	NM	0.038	Yes
	Control	1.00 (2.19)^††^	0.49 (2.19)^††^			

Methacholine PC_20_, the provocation concentration of methacholine causing a 20% fall in FEV_1_; *NM*, not mentioned

### FeNO and methacholine PC_20_

Similar to sputum and blood eosinophils, data of FeNO and methacholine PC_20_ from available studies could not be pooled in meta-analysis. In total, six studies [13, 16, 17, 20, 21, 24] and two studies [13, 24] depicted change of FeNO and methacholine PC_20_, respectively. No significant difference in FeNO was found between CRTH2 antagonists and placebo even in CRTH2 antagonists monotherapy or as add-on treatment to corticosteroids. However, Diamant et al. [24] showed that Setipiprant monotherapy significantly stablized methacholine PC_20_ in stable allergic steroid-free asthma compared to placebo, which was not observed in the study by Singh et al. [13] in stable allergic steroid-naïve asthmatics treated with OC000459 monotherapy.

## Discussion

In our study, we found that CRTH2 antagonists, compared to placebo, significantly improved pre-bronchodilator FEV_1_ (L) and AQLQ scores, reduced ACQ scores and SABA use in adults with asthma, which was also true in the treatment of CRTH2 antagonists monotherapy except for no effect on ACQ scores but improved pre-bronchodilator FEV_1_% predicted and reduced asthma exacerbations. However, CRTH2 antagonists as add-on treatment to corticosteroids did not show any obvious superior advantages over placebo. CRTH2 antagonists monotherapy was associated with lesser adverse events leading to treatment withdrawal, but CRTH2 antagonists, regardless of monotherapy or as add-on treatment to corticosteroids, showed similar incidence of adverse events, severe adverse events, and treatment related adverse events compared with placebo.

Reversible airflow limitation and airway hyperresponsiveness are the key traits in asthma pathophysiology, and FEV_1_, PEF as well as Methacholine PC_20_ are the most widely used parameters to assess asthma severity and control, and predict future risk of asthma exacerbations [26, 27]. Our study found that CRTH2 antagonists monotherapy could significantly improve pre-bronchodilator FEV_1_ and FEV_1_% predicted, which might be attributed to the potential anti-inflammation effects of CRTH2 antagonists [5–7]. As mentioned above, the binding of PGD2 to CRTH2 induces respiratory burst and degranulation of eosinophils as well as increases release of type 2 cytokines, leukotrienes and cationic proteins, which may damage airway epithelia, thus resulting in airway narrowing and development of airway hyperresponsiveness [7, 28–30]. Furthermore, it has been demonstrated that production of type 2 cytokines is associated with greater decline in lung functions [31]. However, in our study, no additional synergistic effects were observed when CRTH2 antagonists were used as add-on treatment to corticosteroids. With consideration of the meta-regression analysis, which indicated no association between pre-bronchodilator FEV_1_ and either asthma severity, concomitant treatment, or treatment duration, the non-superiority of add-on treatment of CRTH2 antagonists to corticosteroids might result from: 1) the difference in CRTH2 antagonists types and doses with various bioavailability, pharmacokinetics and pharmacodynamics; 2) the true benefit of CRTH2 antagonists being covered by the potent effects of concurrent corticosteroids use. For example, Hall et al. [14] also found that in steroids-naïve rather than steroids-on-use asthmatics 400 mg of BI 671800 could improve lung function [25].

In terms of PEF, our pooled and subgroup analysis revealed no improvement of morning and evening PEF in asthmatics with CRTH2 antagonists treatment. Moreover, one study [17] even reported that CRTH2 antagonists together with corticosteroids could reduce morning PEF. The poor relationship between FEV_1_ and PEF might relate to the disassociation of the effect of CRTH2 antagonists on these two parameters [32, 33]. However, the effect of CRTH2 antagonists on PEF should be interpreted cautiously because of the moderate heterogeneity and limited studies included, which is also true for Methacholine PC_20_. Thus, further studies are needed to clarify the effect of CRTH2 antagonists on PEF and Methacholine PC_20_.

It is recommended by GINA that asthma assessment should focus on asthma symptom control and future exacerbations risk reduction [2]. ACQ and AQLQ are both commonly used self-evaluation questionnaires for asthma symptoms and quality of life [34, 35], and our meta-analysis showed that CRTH2 antagonists therapy could reduce ACQ scores and increase AQLQ scores. SABA is one of the most important quick relievers for asthma onset, and the number of its rescue use has already been elucidated to be associated with asthma exacerbations [2]. Our meta-analysis also found that CRTH2 antagonists, either used as monotherapy or add-on therapy to corticosteroids, could reduce SABA use. Asthma exacerbations are associated with the poor asthma control [2] and is the major cause of morbidity and mortality in asthmatics [36]. In our meta-analysis, significant reduction in asthma exacerbations was found in the asthmatics with CRTH2 antagonist monotherapy rather than add-on therapy to corticosteroids. Therefore, based on the above positive findings, CRTH2 antagonists may serve as an efficacious surrogate for corticosteroids and reduce the use or adverse events of corticosteroids. However, future studies are still warranted due to the inconsistent of CRTH2 antagonists types, doses, and durations, as well as the potential heterogeneities and limited studies.

It is reported that sputum or blood eosinophil level is associated with high incidence of asthma attacks, and they are also one of the important markers for asthma phenotyping [37, 38]. Meanwhile, blockade of CRTH2 has been recognized to down-regulate Th2 cytokines production [8], decrease eosinophils release from bone marrow [7, 39], chemotaxis and respiratory burst [28]. Therefore, eosinophil might be a potential indicator for treatment effectiveness and asthma phenotyping may also help to identify the better responsive subgroup. However, inconsistent data from the included studies disabled us to pool in meta-analysis, and our systematic review also showed inconclusive results. FeNO is believed to be an indirect marker for eosinophilic airway inflammation [40], and our systematic review found that CRTH2 antagonists could not decrease FeNO. However, more studies are necessitated before we can draw a clear conclusion because the role of FeNO itself in asthma airway inflammation is still not clarified and controversial findings showed that the specific inhibition of inducible nitric oxide synthase did not affect airway hyperresponsiveness and airway inflammation [41].

In our meta-analysis, we found similar adverse events between CRTH2 antagonists treatment and placebo, and no treatment related severe adverse events and deaths were reported, which indicated a general safety profile of CRTH2 antagonists in the treatment of asthma patients. Although CRTH2 antagonists were reported to cause some adverse events, but most of them were mild and moderate such as nasopharyngitis, headache, asthma, infections and gastrointestinal disorders. However, use with cautions, especially for some elderly patients with concomitant diseases, should always be addressed.

Several potential limitations require consideration in interpreting our study results. First of all, although some parameters, such as pre-bronchodilator FEV_1_, ACQ and AQLQ scores, have been improved in patients with CRTH2 antagonists, the clinical importance of these improvements need to be questioned because they are less than minimal clinical importance difference [35, 42, 43]. Secondly, asthma exacerbations in the trials included were not defined consistently and even not defined explicitly in one trial [22]. Thirdly, a small scale of some studies and limited number of RCTs included in several outcomes analysis may affect the power to explore the real outcome. Finally, the heterogeneities among the studies might cause inaccurate results in some outcomes. Although we have classified the studies into subgroups based on the intervention therapies and we found no statistical heterogeneities in most of the outcomes, but the baseline asthma severity and phenotypes varied among studies, which makes it necessary for further studies to clarify which subgroups of asthmatics can benefit this treatment. Moreover, given the variety of CRTH2 antagonists in selectivity, specificity and affinity, such as the dual affinity of AMG 853 to both DP2 and DP1, the interpretation of our results should also be cautious and it is hard to decide the optimal types of CRTH2 antagonists, dose, and treatment duration. Therefore, future studies involving and dealing with these issues are urgently needed.

## Conclusions

In patients with asthma, CRTH2 antagonists especially being administered as monotherapy were well tolerated and efficacious in improving lung function and quality of life , as well as reducing rescue use of SABA and asthma exacerbations. CRHT2 antagonists might be suitable surrogates for corticosteroids in patients who are contraindicated to steroids treatment or who require steroids limitation to avoid related adverse events. However, further trials are necessitated, particularly in different asthma phenotypes as well as in comparison between CRTH2 antagonists and corticosteroids monotherapy, to identify the potential asthma subgroups with best treatment responses and determine the optimal administration strategy of CRTH2 antagonists.

## Additional file

Additional file 1: **Figure S1.** Begg’s test for publication bias on pre-bronchodilator FEV1 (L). **Figure S2.** Meta-regression plot of mean difference for pre-bronchodilator FEV_1_ (L) predicted by treatment duration. **Figure S3.** Meta-regression plot of mean difference for pre-bronchodilator FEV_1_ (L) predicted by asthma severity. **Figure S4.** Meta-regression plot of mean difference for pre-bronchodilator FEV_1_ (L) predicted by concomitant treatment. **Figure S5.** The effect of CRTH2 antagonists used as monotherapy or add-on therapy versus placebo on pre-bronchodilator FEV_1_% predicted. **Figure S6.** The effect of CRTH2 antagonists used as monotherapy or add-on therapy versus placebo on post-bronchodilator FEV_1_ (L). **Figure S7.** The effect of CRTH2 antagonists used as monotherapy or add-on therapy versus placebo on FVC. **Figure S8.** The effect of CRTH2 antagonists used as monotherapy or add-on therapy versus placebo on severe adverse events. **Figure S9.** The effect of CRTH2 antagonists used as monotherapy or add-on therapy versus placebo on treatment related adverse events. **Figure S10.** The effect of CRTH2 antagonists used as monotherapy or add-on therapy versus placebo on adverse events leading to treatment withdrawal. **Figure S11.** Begg’s test for publication bias on adverse event. **Figure S12.** Meta-regression plot of risk ratio for adverse events predicted by treatment duration. **Figure S13.** Meta-regression plot of risk ratio for adverse events predicted by concomitant treatment. **Figure S14.** Meta-regression plot of risk ratio for adverse events predicted by asthma severity. (DOCX 10650 kb)

## Abbreviations

ACQ: Asthma control questionnaire
AQLQ: Asthma quality of life questionnaire
CI: Confidence interval
CRTH2: Chemoattractant receptor-homologous molecule expressed on Th2 cells
FeNO: Fractional exhaled nitric oxide
FEV_1_: Forced volume in one second
FVC: Forced vital capacity
GINA: Global Initiative for Asthma
MD: Mean differences
Methacholine PC_20_: The provocation concentration of methacholine causing a 20% fall in FEV_1_
PEF: Peak expiratory flow
RCT: Randomized controlled trial
RD: Risk difference
RR: Risk ratio
SABA: Short-acting β_2_ agonists
SD: Standard deviation
