# Supplementary material for: Efficacy and safety of antagonists for chemoattractant receptor-homologous molecule expressed on Th2 cells in adult patients with asthma: a meta-analysis and systematic review
Source: Respir Res. 2018 Nov 9;19:217. doi: 10.1186/s12931-018-0912-y (PMC6230288; doi:10.1186/s12931-018-0912-y)
Supplement: Supplementary file 1 — Figure S1. Begg’s test for publication bias on pre-bronchodilator FEV1 (L). Figure S2. Meta-regression plot of mean difference for pre-bronchodilator FEV1 (L) predicted by treatment duration. Figure S3. Meta-regression plot of mean difference for pre-bronchodilator FEV1 (L) predicted by asthma severity. Figure S4. Meta-regression plot of mean difference for pre-bronchodilator FEV1 (L) predicted by concomitant treatment. Figure S5. The effect of CRTH2 antagonists used as monotherapy or add-on therapy versus placebo on pre-bronchodilator FEV1% predicted. Figure S6. The effect of CRTH2 antagonists used as monotherapy or add-on therapy versus placebo on post-bronchodilator FEV1 (L). Figure S7. The effect of CRTH2 antagonists used as monotherapy or add-on therapy versus placebo on FVC. Figure S8. The effect of CRTH2 antagonists used as monotherapy or add-on therapy versus placebo on severe adverse events. Figure S9. The effect of CRTH2 antagonists used as monotherapy or add-on therapy versus placebo on treatment related adverse events. Figure S10. The effect of CRTH2 antagonists used as monotherapy or add-on therapy versus placebo on adverse events leading to treatment withdrawal. Figure S11. Begg’s test for publication bias on adverse event. Figure S12. Meta-regression plot of risk ratio for adverse events predicted by treatment duration. Figure S13. Meta-regression plot of risk ratio for adverse events predicted by concomitant treatment. Figure S14. Meta-regression plot of risk ratio for adverse events predicted by asthma severity. (DOCX 10650 kb) [file 12931_2018_912_MOESM1_ESM.docx]

Figure S1 Begg’s test for publication bias on pre-bronchodilator FEV1 (L).


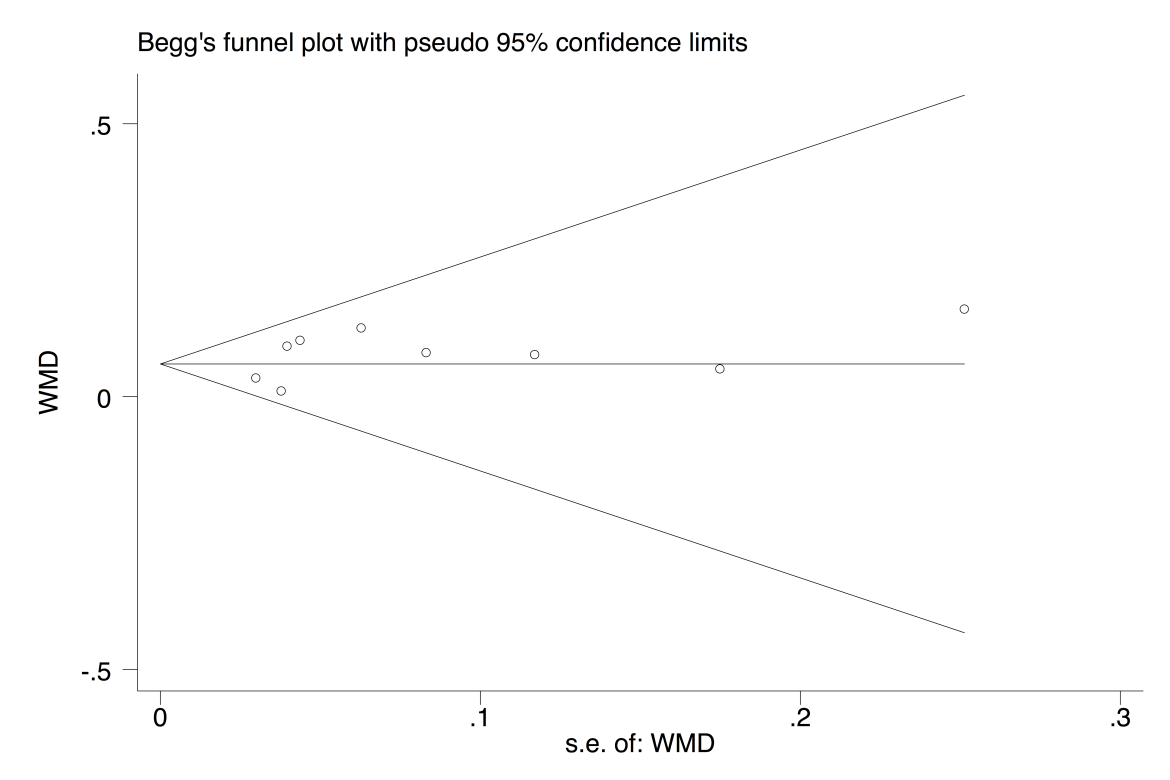


FEV_1_, forced expiratory volume in one second; WMD, weighted mean difference.

Figure S2 Meta-regression plot of mean difference for pre-bronchodilator FEV_1_ (L) predicted by treatment duration.


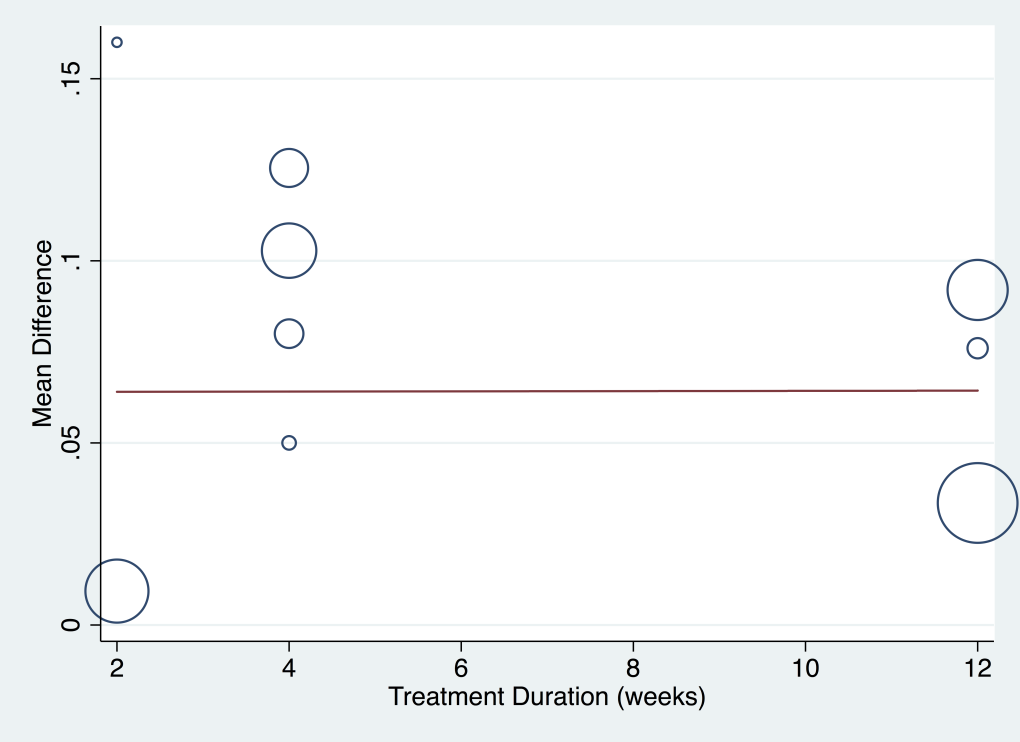


Red line represents point estimates of association between CRTH2 antagonists treatment duration (weeks) and mean difference of pre-bronchodilator FEV_1_ (L). Circles represent studies included in the analysis and their size is proportional to the weights assigned in meta-regression. CRTH2, chemoattractant receptor-homologous molecule expressed on Th2 cells; FEV_1_, forced expiratory volume in one second.

Figure S3 Meta-regression plot of mean difference for pre-bronchodilator FEV_1_ (L) predicted by asthma severity.


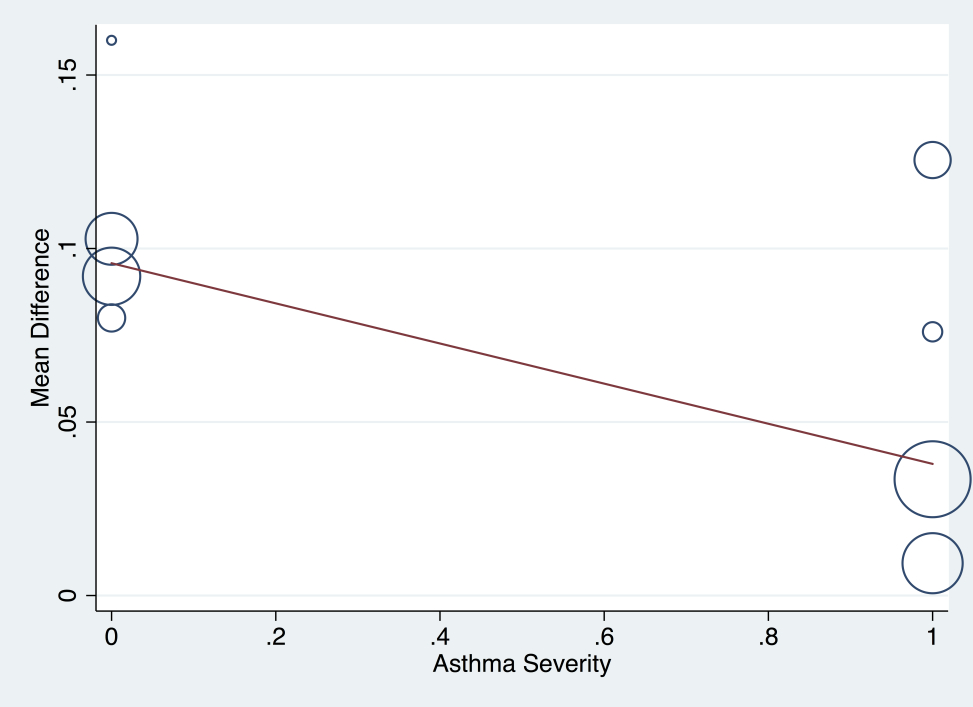


Red line represents point estimates of association between asthma severity and mean difference of pre-bronchodilator FEV_1_ (L) in the treatment of CRTH2 antagonists. Circles represent studies included in the analysis and their size is proportional to the weights assigned in meta-regression. 0 stands for mild, mild-to-moderate and moderate asthma, 1 stands for moderate-to-severe asthma. CRTH2, chemoattractant receptor-homologous molecule expressed on Th2 cells; FEV_1_, forced expiratory volume in one second.

Figure S4 Meta-regression plot of mean difference for pre-bronchodilator FEV_1_ (L) predicted by concomitant treatment.


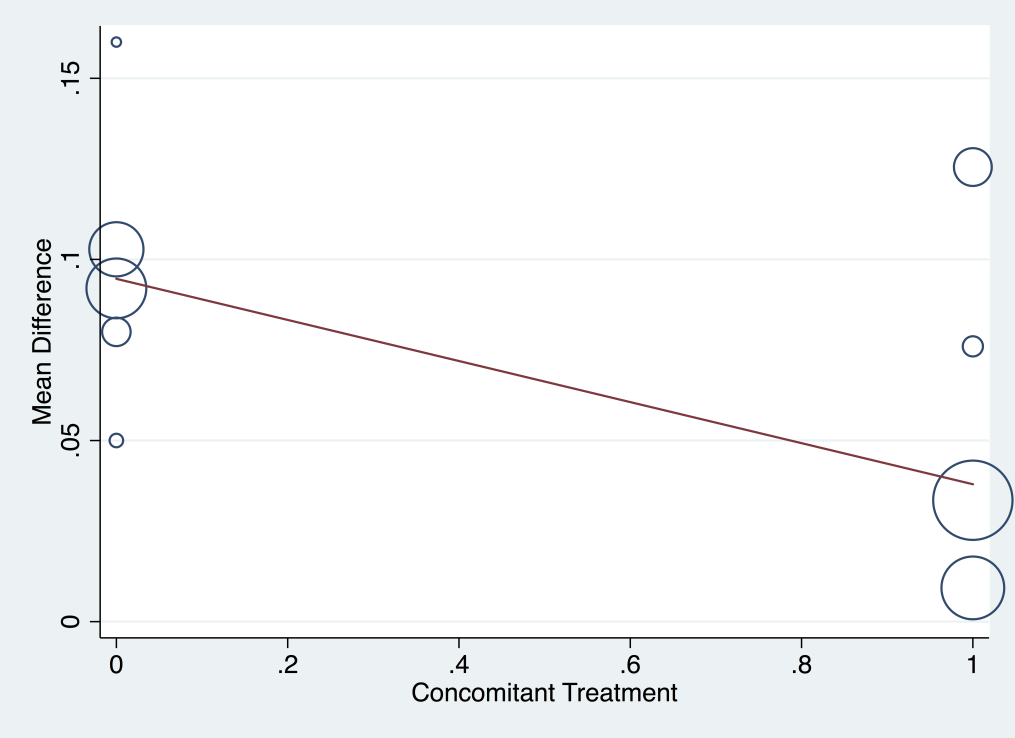


Red line represents point estimates of association between concomitant treatment and mean difference of pre-bronchodilator FEV_1_ (L) in the treatment of CRTH2 antagonists. Circles represent studies included in the analysis and their size is proportional to the weights assigned in meta-regression. 0 stands for CRTH2 monotherapy, 1 stands for CRTH2 antagonists used as add-on to corticosteroids therapy. CRTH2, chemoattractant receptor-homologous molecule expressed on Th2 cells; FEV_1_, forced expiratory volume in one second.

Figure S5 The effect of CRTH2 antagonists used as monotherapy or add-on therapy versus placebo on pre-bronchodilator FEV_1_% predicted.


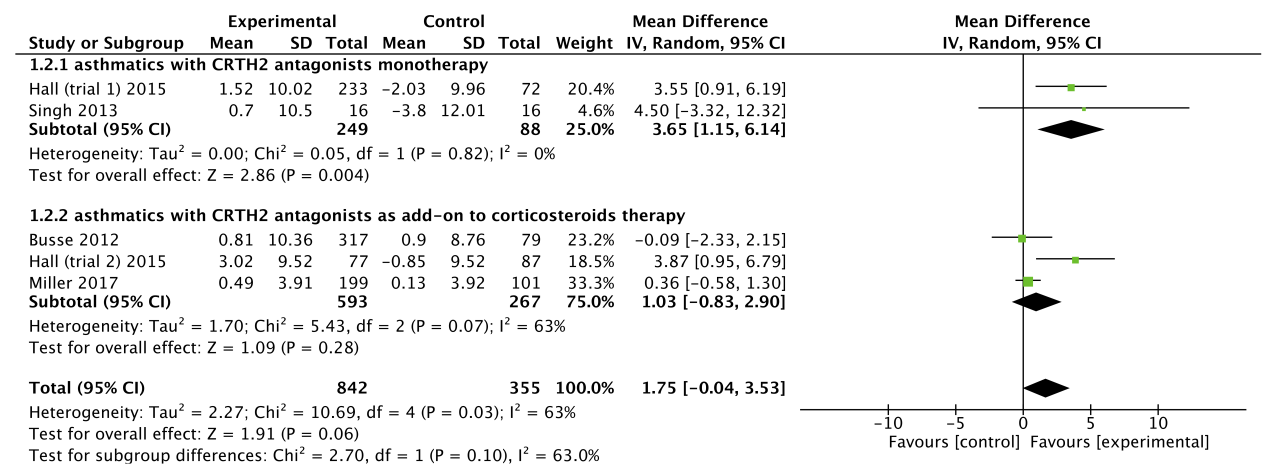


CI, confidential interval; CRTH2, chemoattractant receptor-homologous molecule expressed on Th2 cells; FEV_1_, forced expiratory volume in one second; SD, standard deviation

Figure S6 The effect of CRTH2 antagonists used as monotherapy or add-on therapy versus placebo on post-bronchodilator FEV_1_ (L).


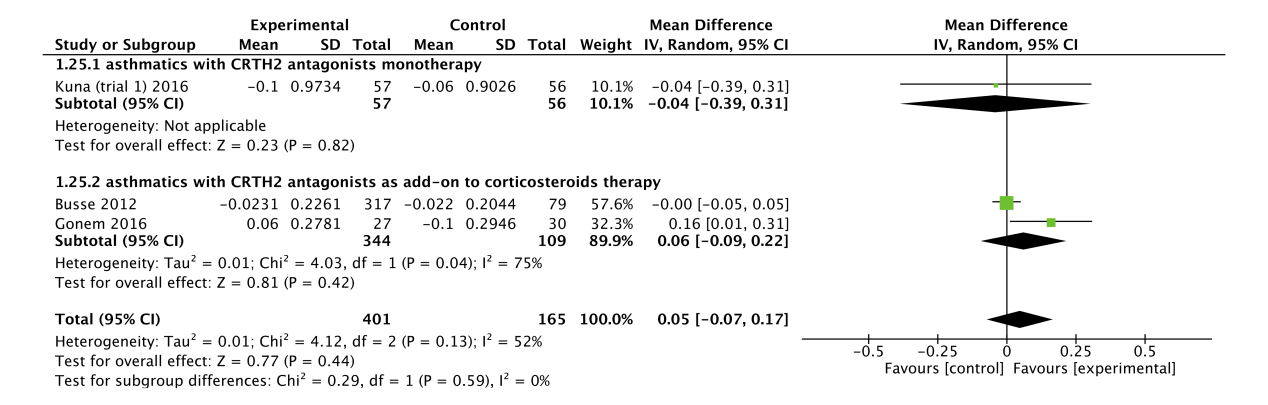


CI, confidential interval; CRTH2, chemoattractant receptor-homologous molecule expressed on Th2 cells; FEV_1_, forced expiratory volume in one second; SD, standard deviation.

Figure S7 The effect of CRTH2 antagonists used as monotherapy or add-on therapy versus placebo on FVC.


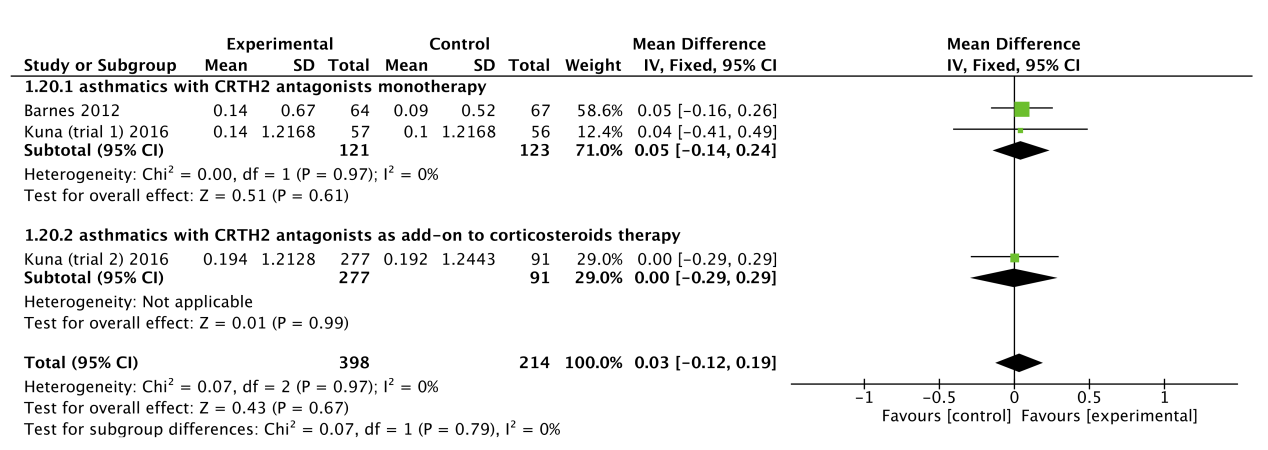


CI, confidential interval; CRTH2, chemoattractant receptor-homologous molecule expressed on Th2 cells; FVC, forced vital capacity; SD, standard deviation.

Figure S8 The effect of CRTH2 antagonists used as monotherapy or add-on therapy versus placebo on severe adverse events.


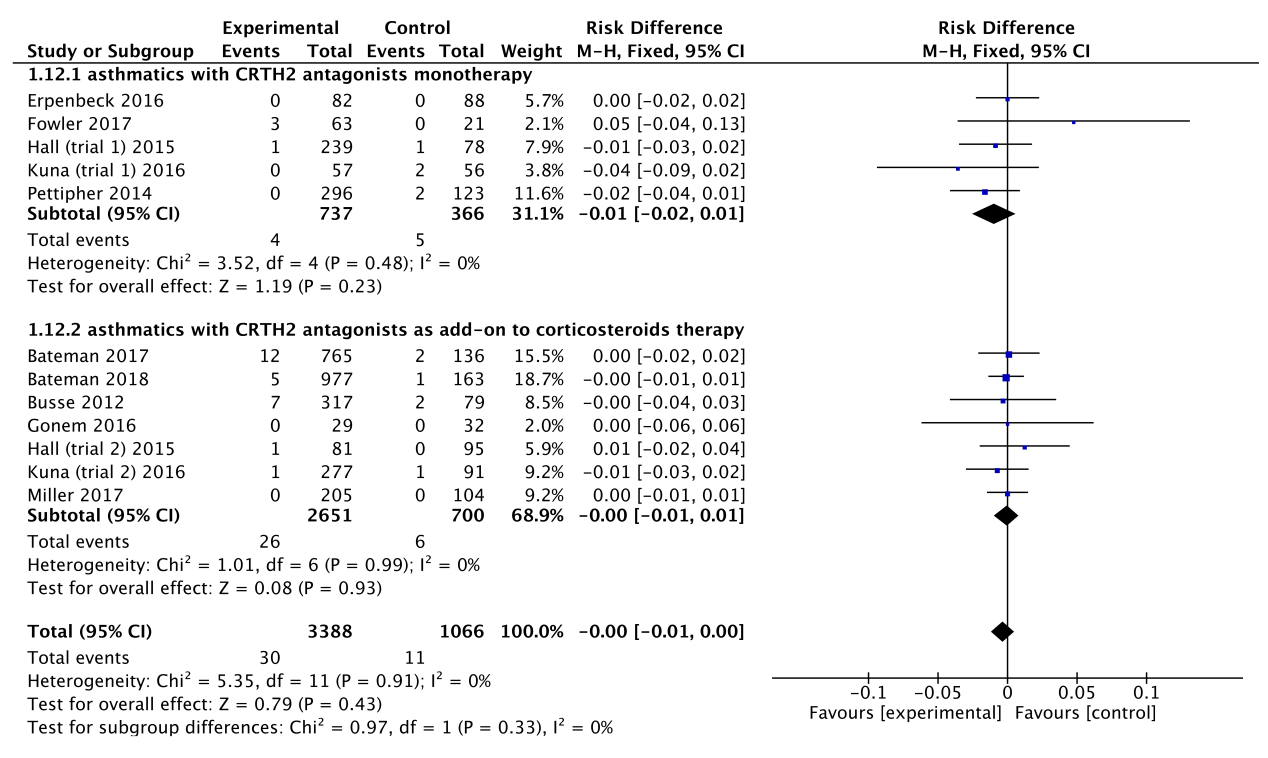


CI, confidential interval; CRTH2, chemoattractant receptor-homologous molecule expressed on Th2 cells.

Figure S9 The effect of CRTH2 antagonists used as monotherapy or add-on therapy versus placebo on treatment related adverse events.


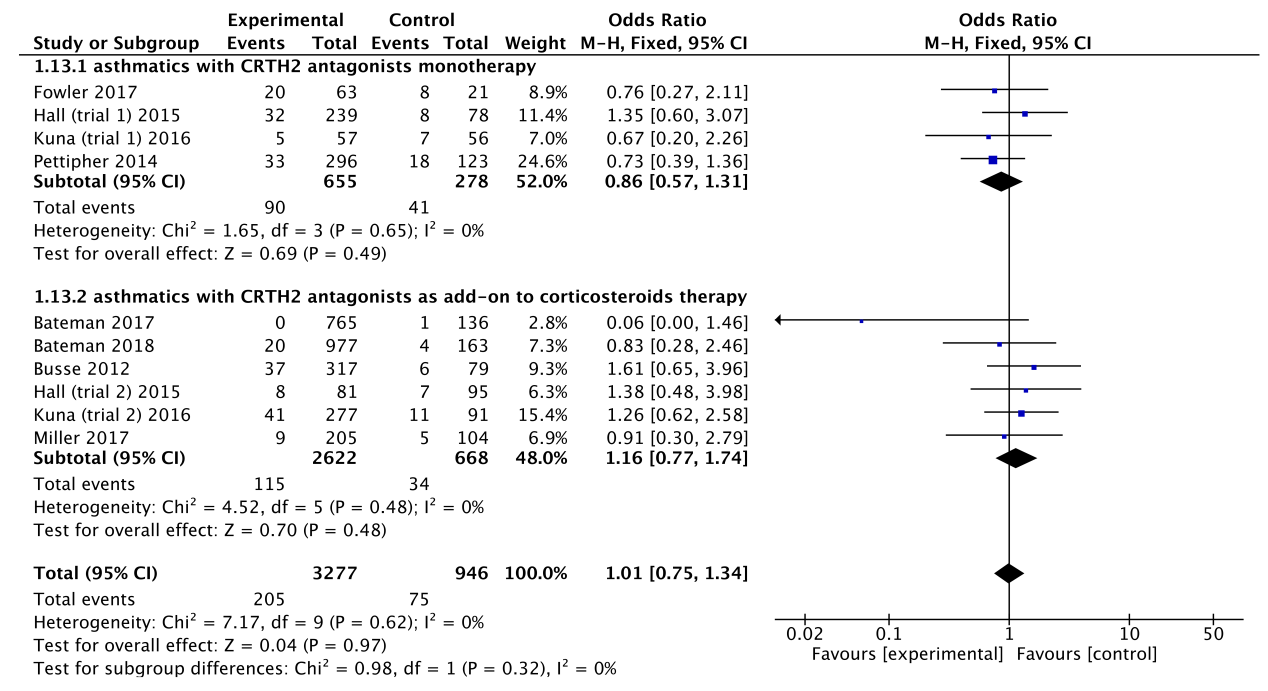


CI, confidential interval; CRTH2, chemoattractant receptor-homologous molecule expressed on Th2 cells.

Figure S10 The effect of CRTH2 antagonists used as monotherapy or add-on therapy versus placebo on adverse events leading to treatment withdrawal.


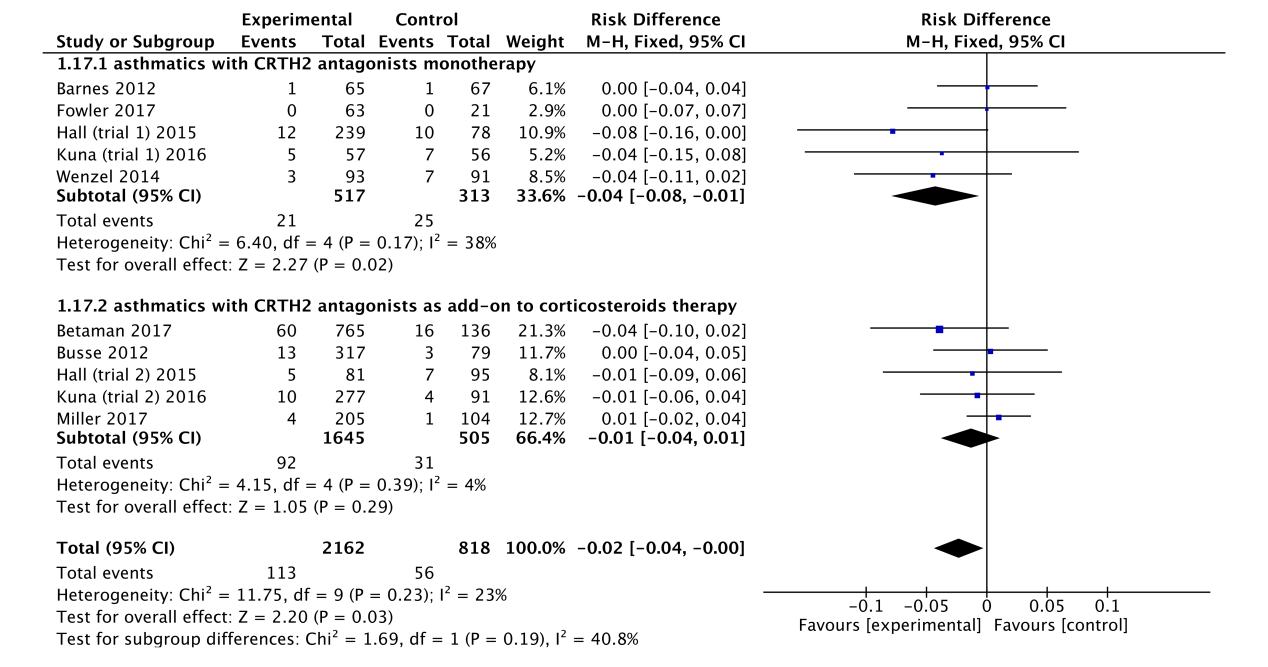


CI, confidential interval; CRTH2, chemoattractant receptor-homologous molecule expressed on Th2 cells.

Figure S11 Begg’s test for publication bias on adverse event
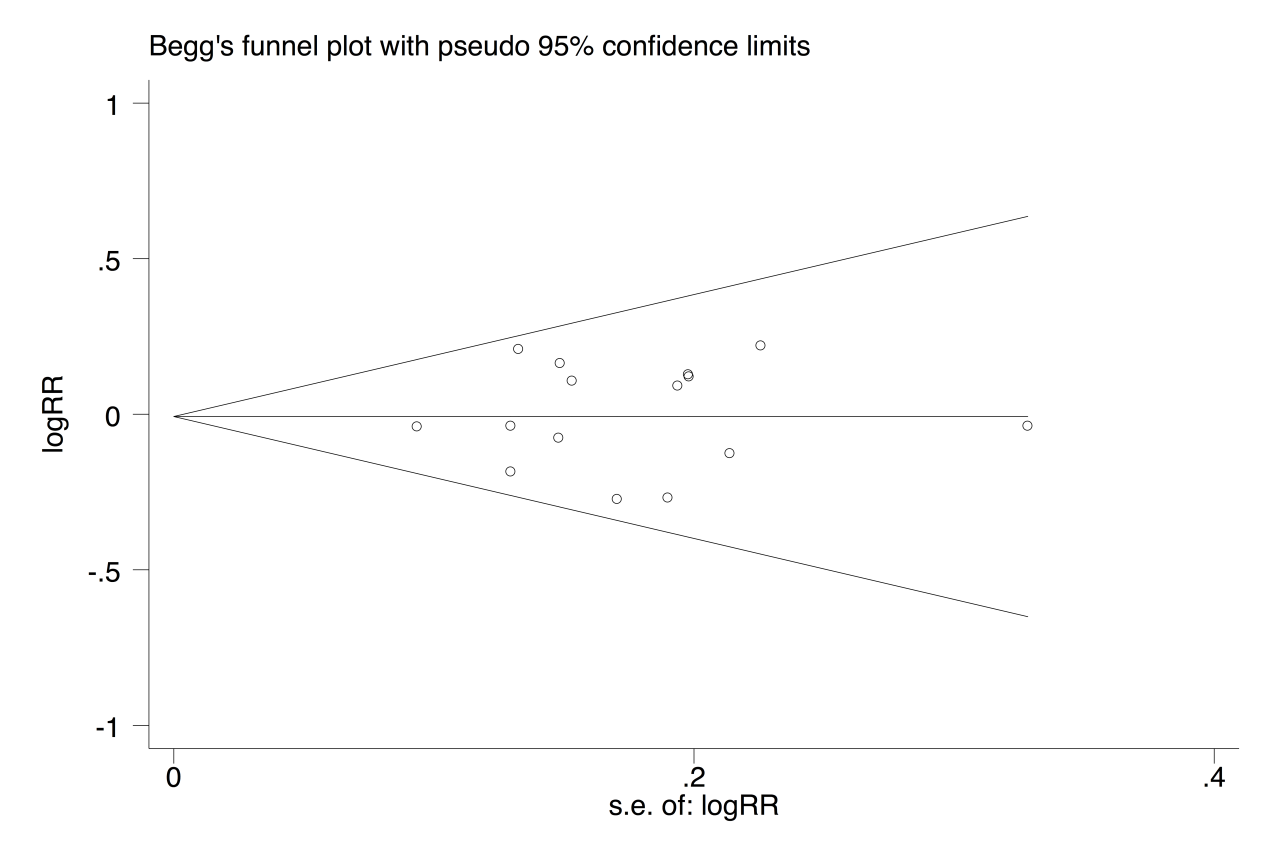
RR, risk ratio.

Figure S12 Meta-regression plot of risk ratio for adverse events predicted by treatment duration.


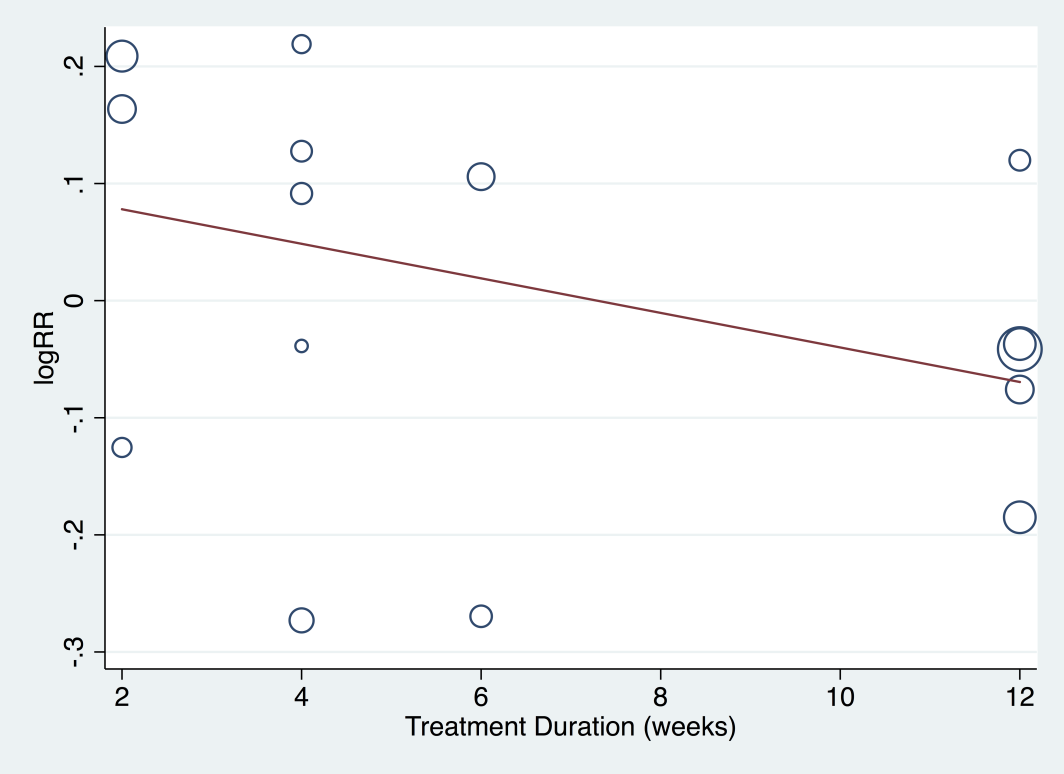


Red line represents point estimates of association between CRTH2 antagonists treatment duration (weeks) and risk ratio of adverse events. Circles represent studies included in the analysis and their size is proportional to the weights assigned in meta-regression. The vertical axis is on a log scale. CRTH2, chemoattractant receptor-homologous molecule expressed on Th2 cells; RR, risk ratio.

Figure S13 Meta-regression plot of risk ratio for adverse events predicted by concomitant treatment.


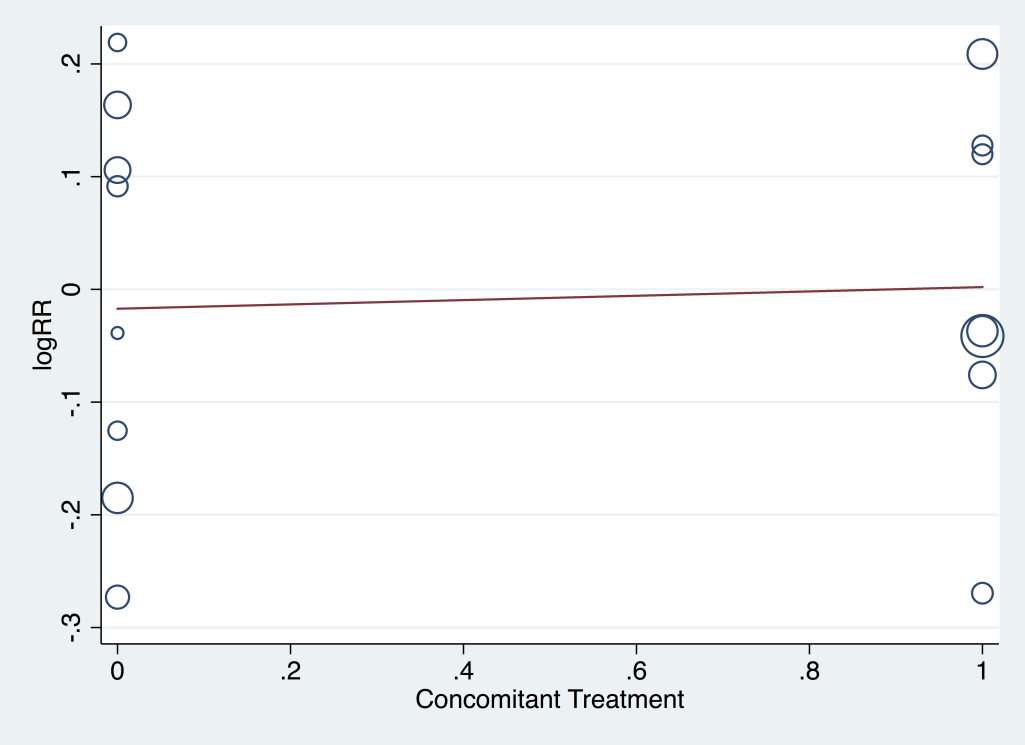


Red line represents point estimates of association between concomitant treatment and risk ratio of adverse events. Circles represent studies included in the analysis and their size is proportional to the weights assigned in meta-regression. The vertical axis is on a log scale. 0 stands for CRTH2 antagonists monotherapy, 1 stands for CRTH2 antagonists used as add-on to corticosteroids therapy. CRTH2, chemoattractant receptor-homologous molecule expressed on Th2 cells; RR, risk ratio.

Figure S14 Meta-regression plot of risk ratio for adverse events predicted by asthma severity.


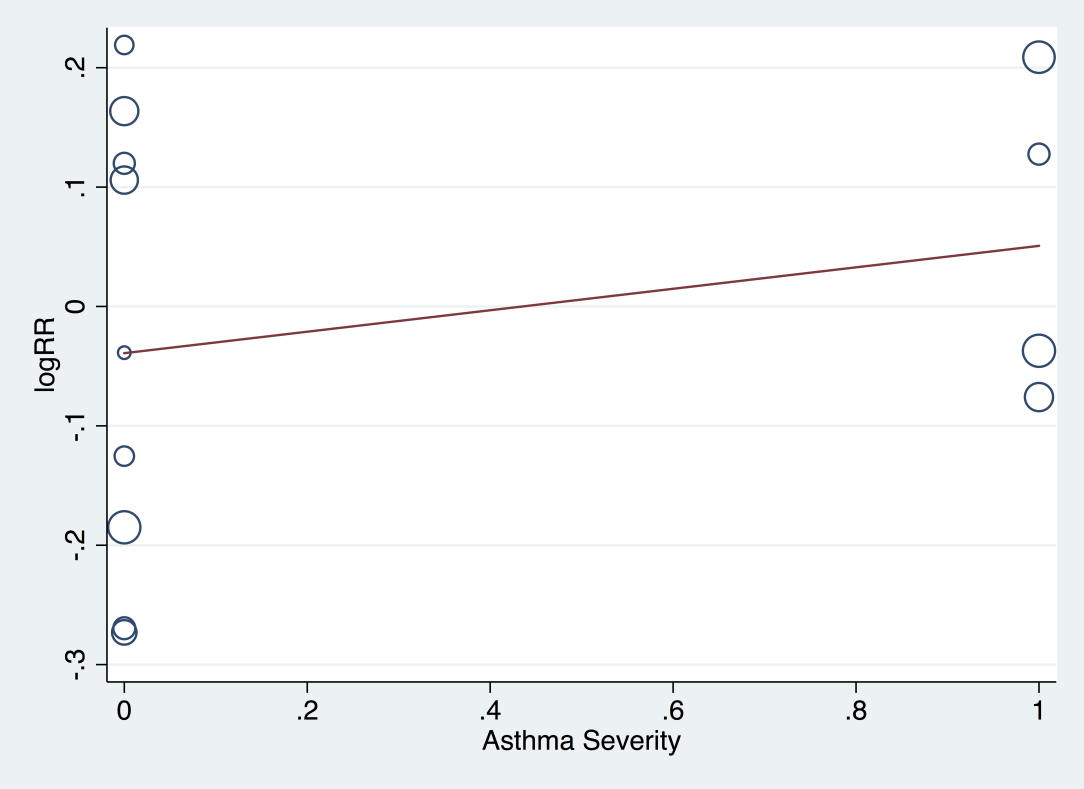


Red line represents point estimates of association between asthma severity and risk ratio of adverse events. Circles represent studies included in the analysis and their size is proportional to the weights assigned in meta-regression. The vertical axis is on a log scale. 0 stands for mild or mild-to-moderate asthma, 1 stands for moderate-to-severe asthma. RR, risk ratio
